# Peptide Bispecifics Inhibiting HIV-1 Infection by an Orthogonal Chemical and Supramolecular Strategy

**DOI:** 10.1021/acs.bioconjchem.3c00314

**Published:** 2023-09-04

**Authors:** Dominik Schauenburg, Fabian Zech, Astrid Johanna Heck, Pascal von Maltitz, Mirja Harms, Siska Führer, Nico Alleva, Jan Münch, Seah Ling Kuan, Frank Kirchhoff, Tanja Weil

**Affiliations:** † Max-Planck Institute for Polymer Research, Ackermannweg 10, 55128 Mainz, Germany; ‡ Institute of Molecular Virology, Ulm University Medical Center, Meyerhofstr. 1, 89081 Ulm, Germany

## Abstract

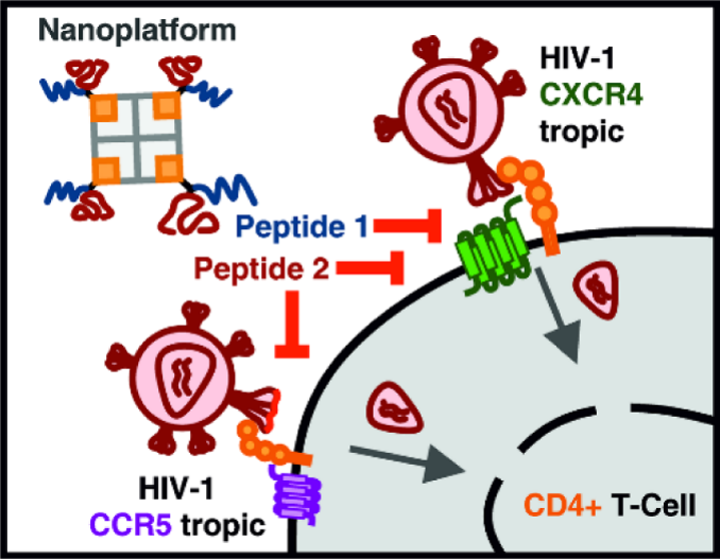

Viral infections pose a significant threat to human health, and effective antiviral strategies are urgently needed. Antiviral peptides have emerged as a promising class of therapeutic agents due to their unique properties and mechanisms of action. While effective on their own, combining antiviral peptides may allow us to enhance their potency and to prevent viral resistance. Here, we developed an orthogonal chemical strategy to prepare a heterodimeric peptide conjugate assembled on a protein-based nanoplatform. Specifically, we combined the optimized version of two peptides inhibiting HIV-1 by distinct mechanisms. Virus-inhibitory peptide (VIRIP) is a 20 amino acid fragment of α1-antitrypsin that inhibits HIV-1 by targeting the gp41 fusion peptide. Endogenous peptide inhibitor of CXCR4 (EPI-X4) is a 16-residue fragment of human serum albumin that prevents HIV-1 entry by binding to the viral CXCR4 co-receptor. Optimized forms of both peptides are assembled on supramolecular nanoplatforms through the streptavidin–biotin interaction. We show that the construct consisting of the two different peptides (SAv-VIR-102C9-EPI-X4 JM#173-C) shows increased activity against CCR5- and CXCR4-tropic HIV-1 variants. Our results are a proof of concept that peptides with different modes of action can be assembled on nanoplatforms to enhance their antiviral activity.

## Introduction

Viral diseases pose substantial threats to public health, socioeconomic stability, and global economic structures, as vividly underscored by the recent SARS-CoV-2 pandemic. Additionally, other pandemic pathogens, like HIV-1, remain inadequately controlled, with approximately 1.7 million new HIV-1 infections and ∼700,000 AIDS-related deaths reported for 2020.^1^ Increasing drug resistance further exacerbates the challenges faced by current antiretroviral treatment strategies. In addition, effective and specific drugs are only available for a very limited number of viral pathogens^2,3^ underscoring the urgent need for novel therapeutic interventions. Most antiviral drugs target viral enzymes to inhibit viral replication.^2^ This requires cellular uptake, which increases the potential for adverse effects. Consequently, therapeutic agents designed to block viral entry into cells provide a promising approach. The process of viral infection is multistage, involving attachment, anchoring, fusion, and eventual entry into host cells, each step offering targets for inhibitory agents. Furthermore, many viruses rely on multiple cellular receptors for infection, which also present potential intervention points. For instance, the initial step in HIV-1 replication involves the attachment of the viral envelope glycoprotein gp120 to the cellular CD4 receptor. This attachment triggers conformational changes that allow gp120 to bind to the CCR5 or CXCR4 co-receptors, subsequently allowing the insertion of the fusion peptide of the viral transmembrane protein gp41 into the target cell membrane. This sequence concludes with the formation of a six-helix bundle, pulling the viral and cellular membranes together to achieve fusion. Essentially all HIV-1 variants are critically dependent on CCR5 or CXCR4 for infection. CCR5 is critical for HIV-1 transmission and used during chronic infection, while CXCR4- and/or dual-tropic viral variants emerge in up to 50% AIDS patients and are associated with poor prognosis.^4,5^ All of these forms of HIV-1 may coexist in infected individuals and need to be targeted for effective therapy and to prevent resistance.^5^

**Figure 1:**
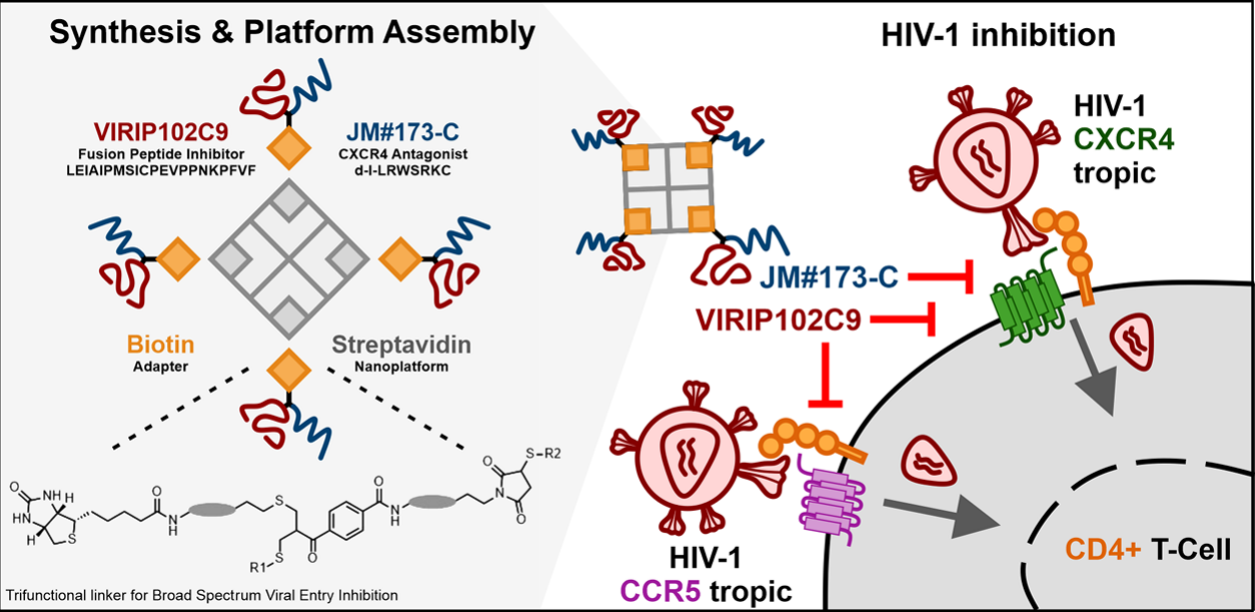
Overview showing the design of the linker for the synthesis of the bispecific VIR-102C9/EPI-X4 JM#173-C and a representation of the antiviral activity of the tetravalent VIR-102C9/EPI-X4 JM#173-C assembled on streptavidin.

**Figure 2:**
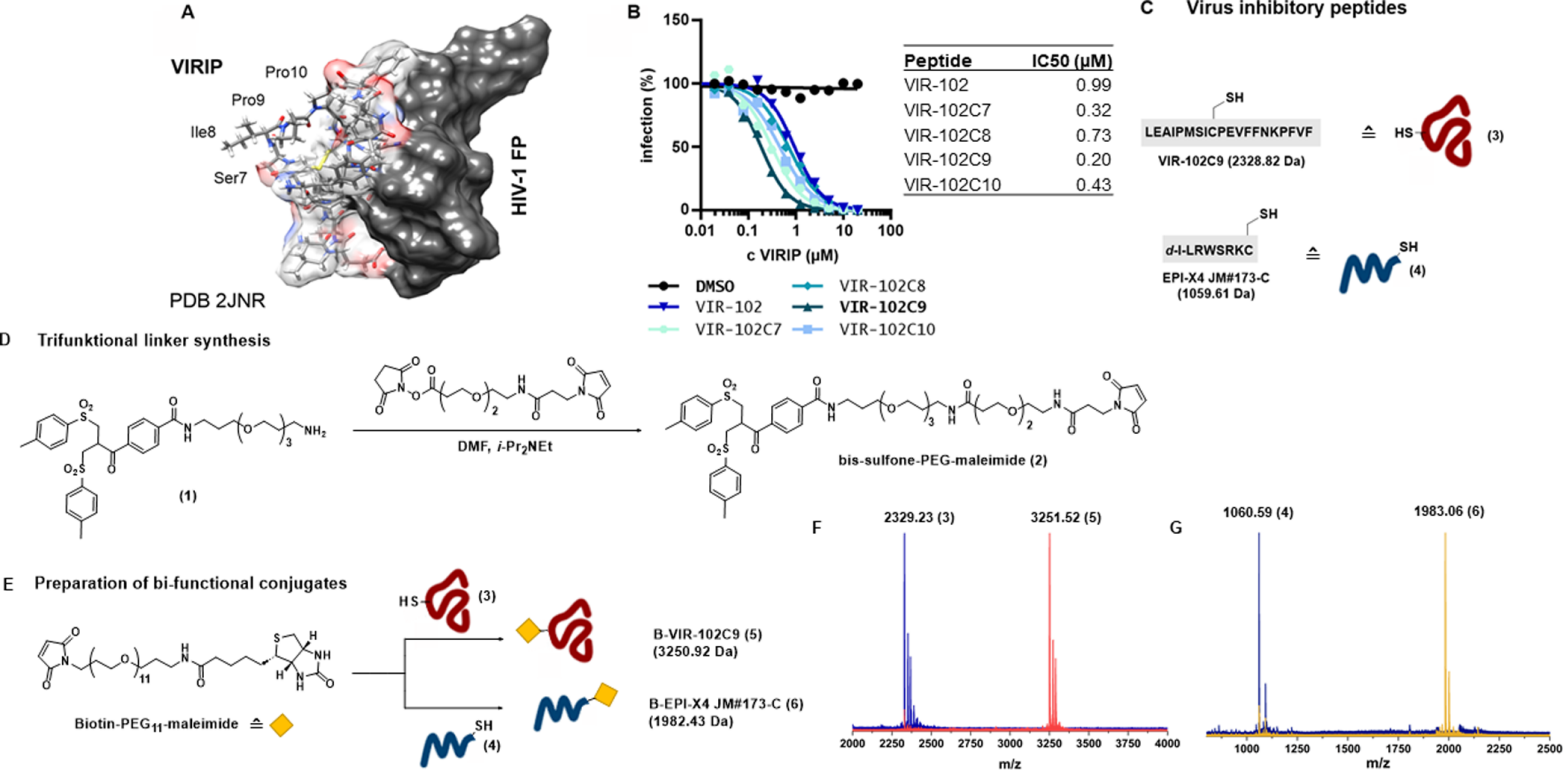
(A) NMR structure of VIR-165 binding the HIV-1 fusion peptide (PDB 2JNR).^17^ VIR-165 positions 7–10 are highlighted. Image is created using UCSF Chimera 1.13.1.^23^ (B) Inhibition of wild-type HIV-1 NL4-3 by single-cysteine VIRIP derivatives. (C) Single letter code and molecular weight of VIR-102C9 (**3**) and EPI-X4 JM#173-C (**4**). (D) Synthesis of the linker bis-sulfone-PEG-maleimide (**2**). (E) Bioconjugation of B-VIR-102C9 (**5**) and B-EPI-X4 JM#173-C (**6**) conjugate. (F) MALDI-TOF spectrum of unconjugated (blue) and biotinylated VIR-102C9 peptide (red). (G) MALDI-TOF spectrum of unconjugated (blue) and biotinylated EPI-X4 JM#173-C peptide (orange). Full spectra of **5** and **6** are available in the SI.

Two entry inhibitors have so far been approved for clinical treatment of HIV-1 infection: Maraviroc (brand name Selzentry) blocks the CCR5 co-receptor on the surface of the host cell but is inactive against HIV-1 strains using CXCR4 for viral entry.^6^ The peptidic fusion inhibitor enfuvirtide (brand name Fuzeon) binds to helical regions in the viral gp41 and prevents six-helix bundle formation required for fusion of the viral and host cell membranes.^7^ Additional co-receptor antagonists and fusion inhibitors have been suggested as possible therapeutic candidates. For example, derivatives of the endogenous peptide inhibitor of CXCR4 (EPI-X4), a 16 amino acid fragment of human serum albumin, act as highly specific CXCR4 antagonists and efficiently inhibit CXCR4 (X4)-tropic HIV-1 strains (Figure 1).^8,9^ Recently, optimized variants of EPI-X4 have been developed, e.g., the seven amino acid EPI-X4 JM#173, which is stable in blood plasma for more than 8 h.^10^ Optimized EPI-X4 derivatives show promise as therapeutic agents for CXCR4-linked diseases, exhibiting anti-inflammatory and anticancer functions in preclinical mouse models.^11,12^ Thus, they are currently further developed for therapeutic applications.^13−16^ VIRIP is the only known inhibitor for the gp41 fusion peptide and prevents anchoring of the virus into the cellular membrane. It consists of 20 amino acids corresponding to the *C*-proximal region of α1-antitrypsin (Figure 2A).^17^ VIRIP-based inhibitors are active against all HIV-1 variants including multiresistant strains due to their distinct mode of action.^17−19^

Intravenous infusion of the optimized VIRIP derivative (VIR-576) reduced the mean plasma viral load by up to 98% without causing severe adverse effects.^18^ In addition, it has been demonstrated that VIRIP-based inhibitors pose a very high barrier to HIV-1 resistance.^19^ However, monotherapy with VIR-576 showed fast clearances and required infusion of high doses of the peptide.^17,18^ Altogether, HIV-1 entry can be targeted by agents that block CD4 receptor or CXCR4 and CCR5 co-receptor engagement, as well as steps involved in membrane fusion. Combining antiretroviral peptides with different modes of action may enhance their potency,^20^ prevent the development of drug resistance, and increase the bioavailability and in vivo half-life due to their enlarged size and combined action. While solid-phase peptide synthesis or native chemical ligation can be used to combine two different peptide sequences, there are limitations. For example, spacers such as poly(ethylene glycol) could be required to ensure that the active amino acids are sufficiently extended and both peptide sequences remain exposed to address the receptors or binding to particles. In other instances, extension of the second peptide sequence from an internal amino acid could be required where the N- or C-termini are critical for activity.^21,22^

To overcome these limitations and generate new antiviral peptide bispecifics, we devised a pH-controlled, stepwise chemical conjugation strategy to prepare and assemble optimized versions of the EPI-X4 derivative JM#173 and the anchoring inhibitor VIRIP (Figure 1). As a proof of concept, we prepared a streptavidin hybrid that contains four copies of the bispecific EPI-X4 JM#173-C and the VIRIP variant 102C9. We demonstrate that this construct inhibits HIV-1 in nanomolar concentrations and shows enhanced activity against CCR5 (R5)- and CXCR4-tropic HIV-1.

## Results and Discussion

### Design of Mono-Peptide and Dipeptide Antiviral Conjugates

To enable the assembly of two antiviral peptides to a supramolecular protein platform, i.e., streptavidin (SAv), we had to further include a biotin group (B) that allows binding to four pockets in tetrameric SAv. Thus, a linker with three sites for chemical functionalization was required. To ensure ease of synthesis of the peptide sequences and minimal influence on the bioactivity of the antiviral peptides, a single cysteine was introduced into each of the peptide sequences. As the N- and C-termini are important for the antiviral activity of VIRIP,^17^ we screened a series of variants with internal cysteines (Figure 2B,C) and selected VIR-102C9 (**3**) with the lowest IC50 (0.20 μM) for further study. In comparison, EPI-X4 derivatives interact with CXCR4 via the seven N-terminal amino acid residues.^11^ Thus, to maintain CXCR4 binding and antiviral activity after conjugation, a C-terminal cysteine was incorporated into EPI-X4 JM#173 and termed JM#173-C, (**4**, Figure 2C). One of the major challenges is to ensure selectivity in a sequential manner with the different thiol-containing peptides.^24^ Specifically, the linker requires three reactive sites for successive Michael additions of natural amino acids (cysteine sidechains) and biotin thiol in a chemo-selective fashion. Thus, we designed a linker, which allows pH-controlled reaction of different thiol-containing molecules of interest (Figure 2D). As a first thiol-reactive group, we chose the well-known maleimide reagent, which can undergo Michael addition even under slightly acidic reaction conditions, due to its high reactivity.^25,26^ As a second chemical handle, we applied a bis-sulfone that is activated only in slightly alkaline condition, for disulfide re-bridging^27^ or for two successive thiol conjugations (Figure 2D).^28^

**Figure 3:**
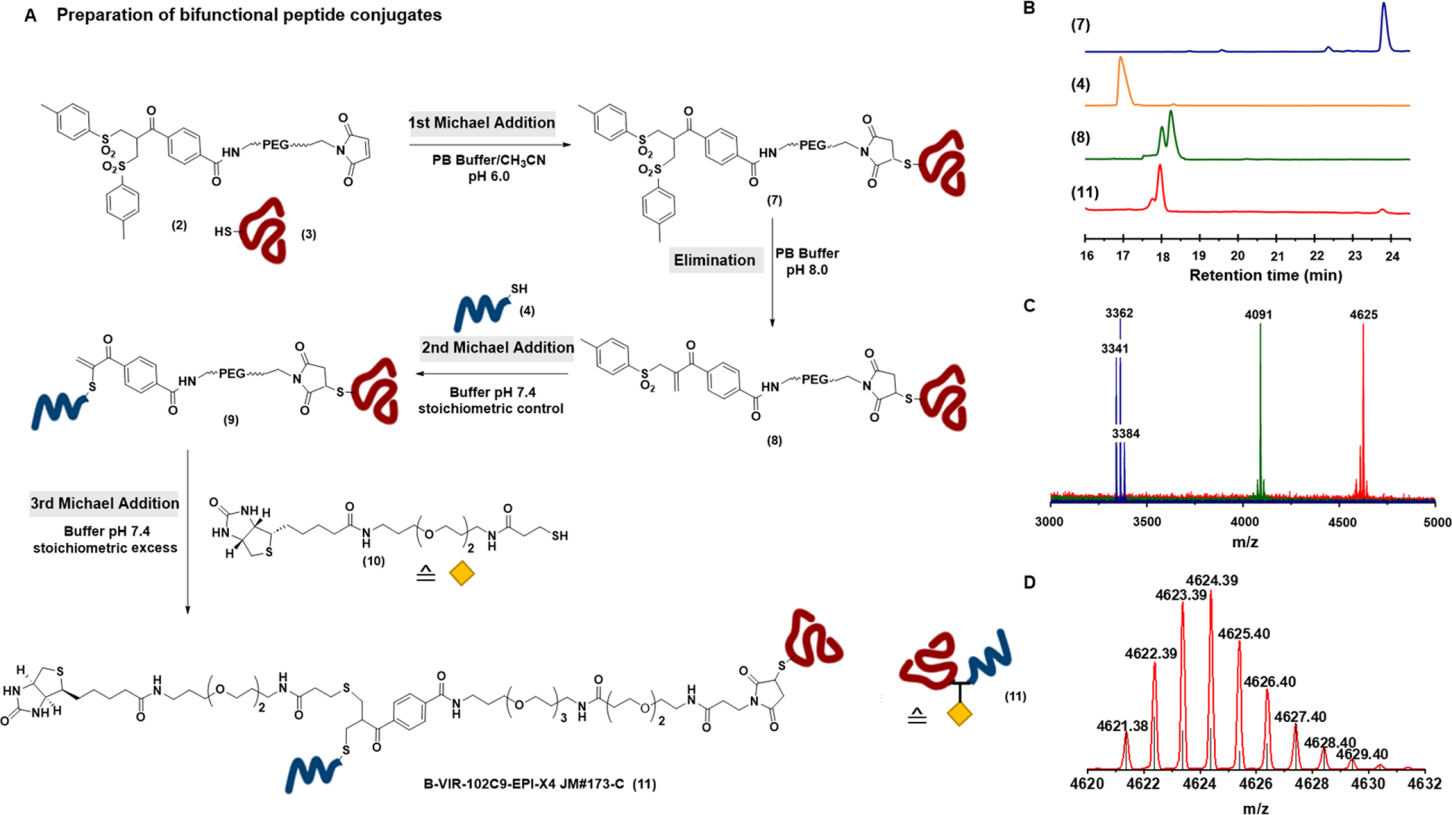
(A) Bioconjugation of B-VIR-102C9-EPI-X4 JM#173-C peptide conjugate (**11**). (B) HPLC spectrum of VIR-102C9 bis-sulfone (**7**), EPI-X4 JM#173-C peptide (**4**), VIR-102C9-EPI-X4 JM#173-C vinyl thioether as a racemic mixture (**9**) and B-VIR-102C9-EPI-X4 JM#173-C conjugate (**11**). (C) MALDI-TOF spectrum of VIR-102C9 bis-sulfone (**7**) (blue), VIR-102C9-EPI-X4 JM#173-C vinyl thioether (**9**) (green), and B-VIR-102C9-EPI-X4 JM#173-C conjugate (**11**) (red). Full spectra of **7, 9**, and **11** are available in the SI. (D) Isotopic pattern of deconvoluted TOF MS ESI spectrum in positive mode of B-VIR-102C9-EPI-X4 JM#173-C (**11**). Deconvoluted spectrum for **11** showing molecular weight. Exact mass determined for m/z = [M+5H]^+^ calc: 925.823, found 925.2822.

We began our studies with the preparation of bifunctional conjugates (**5** and **6**) consisting of the individual antiviral peptide (VIR-102C9, **3** or EPI-X4 JM#173-C, **4**) and a biotin group for assembly. Biotinylation of the peptides was performed using a commercially available biotin-PEG_11_-maleimide (see Figure 2E) under neutral, buffered conditions. We obtained bifunctional conjugates B-VIR-102C9 (**5**) and B-EPI-X4 JM#173-C (**6**) in 62 and 67% yields, respectively. The peptides were identified by MALDI-ToF mass spectrometry through their *m*/*z* at 3252 and 1983 [M + H]^+^, respectively. Monopeptides **5** and **6** were further assembled to the tetrameric biotin-binding protein (SAv) and used as controls for comparison with the bifunctional construct derived from the newly designed B-VIR-102C9-EPI-X4 JM#173-C (**11**, see Figure 3A).

To allow multimerization of different antiviral peptides on SAv, we aimed to conjugate the HIV-1 fusion peptide inhibitor VIR-102C9 (**3**) and the CXCR4 antagonist EPI-X4 JM#173-C (**4**) to our newly designed linker molecule (**2**). In the first step, we conjugated VIR-102C9 selectively to the maleimide under slightly acidic conditions (pH 6.0) to afford VIR-102C9 bis-sulfone (**7**) and VIR-102C9 allyl sulfone (**8**) after HPLC purification. β-Ketosulfones are prone to undergo elimination reactions under strongly basic conditions to yield α,β-unsaturated carbonyl compounds.^29^ A small peak was observed in the chromatogram which could be due to a trace amount of elimination of the sulfinic acid in acidic pH. However, due to the fast reaction rate of the maleimide-thiol addition, this will not have a substantial effect on the chemoselectivity.^27^ Furthermore, the products were purified by HPLC. Thereafter, **7** was incubated at pH 8.0 enabling the elimination of the first *p*-toluoyl sulfinic acid to gain the thiol-reactive allyl sulfone (**8**). The second cysteine-containing peptide (EPI-X4 JM#173-C, **4**) was added to the mixture resulting in conjugate addition and, sequential elimination of the second *p*-toluoyl sulfinic acid, to afford VIR-102C9-EPI-X4 JM#173-C vinyl thioether (**9**). This generates another Michael acceptor, to which was added a biotin-PEG_3_-thiol (**10**). The whole course of the successive reactions was followed with HPLC (Figure 3B). After this three-step one-pot reaction, we isolated the bifunctional peptide conjugate (**11**, B-VIR-EPI-X4 JM#173-C) for supramolecular protein hybrids with precise stoichiometry with an overall yield of 14%. The identity was confirmed by HR-ESI-MS (Figure 3D).

### Supramolecular Assembly of Biotinylated Peptides onto Protein Platforms

**Figure 4:**
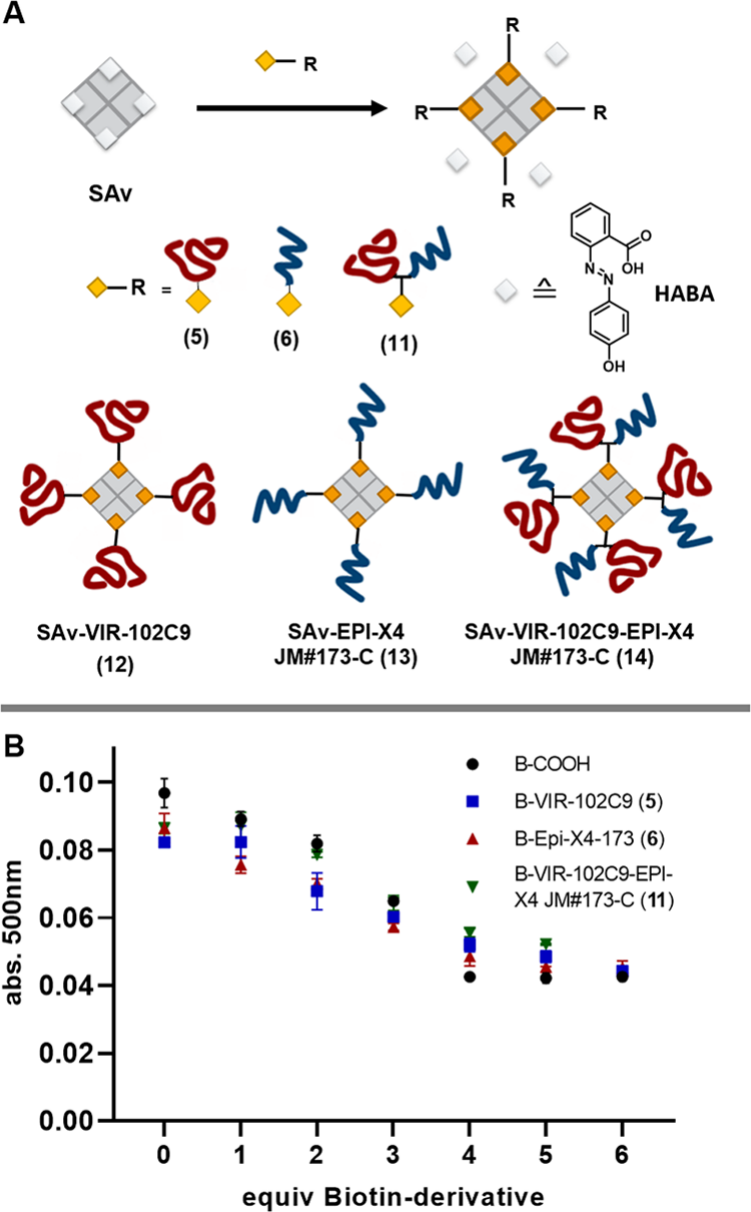
(A) Schematic representation of the supramolecular assembly of biotinylated peptides onto the streptavidin (SAv) platform. (B) Absorbance at 500 nm plotted against biotin and biotinylated peptides to determine stoichiometry required to saturate biotin-binding pockets on SAv. (For **11**, a maximum of five equivalents were used in the HABA assay).

We aimed to investigate the bioactivity of the bispecific antiviral peptides in one supramolecular platform. Due to their strong binding affinity to biotin (*k*_D_ = 10^–15^ M),^30^ the well-documented bio-applicability,^31,32^ and their ability to bind up to four equivalents of the native ligand, we chose the avidin-like protein streptavidin as a supramolecular platform. First, we investigated the number of biotinylated conjugates required to saturate the four binding pockets per SAv, in comparison to its native ligand biotin. We applied the 2(4-hydoxyphenylazo)benzoic acid (HABA)-assay for this purpose (see Figure 4A,B). The diazo-compound HABA binds to the biotin pockets of avidin-like proteins with lower affinity than biotin itself (*k*_D_ = 5 × 10^–6^ M).^33^ Thus, it is replaced by the natural ligand, if present.^33,34^ Since the complex of HABA with SAv shows characteristic absorbance at 500 nm, upon saturation of all four binding pockets with biotin, the absorbance intensity at 500 nm does not decrease further.^33−35^ For the HABA assay, we examined the displacement using increasing equivalents of the biotinylated peptides **5**, **6**, and **11** (Figure 4B). Four equivalents of biotinylated peptides (**5**, **6**, or **11**) per SAv were required for the assembly. Supramolecular assemblies for subsequent biological investigations were performed by mixing **5**, **6**, or **11** with SAv in phosphate buffer at physiological pH, followed by ultraspin filtration purification. In this way, SAv-VIR-102C9 (**12**), SAv-EPI-X4 JM#173-C (**13**), and SAv-VIR-102C9-EPI-X4 JM#173-C (**14**) were generated, respectively. The height tomographic image of SAv-VIR-102C9-EPI-X4 JM#173-C (**14**) was obtained using atomic force microscopy (AFM). AFM shows particles with a maximum height of 8 nm (SI Figure S14). The average height was determined to be 5.5 ± 0.8 nm and showed particle homogeneity (SI Figure S14, Table S1), similar to SAv protein constructs reported in the literature.^36^ Notably, we did not observe larger aggregates.

### Effect of SAv-Coupled VIR-102C9 and EPI-X4 JM#173-C Derivatives on CXCR4- and CCR5-Tropic HIV Infection

**Figure 5:**
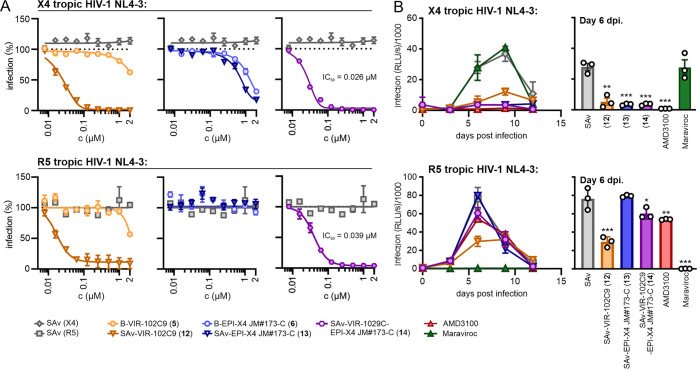
Antiviral activity of single- and multivalent VIR-102C9/EPI-X4 JM#173-C conjugates. Concentrations indicate the molarity of the tested biotin-conjugated peptides or of the SAv conjugates with four copies of mono- or bispecific peptides respectively. (A) TZM-bl cells were pretreated with the indicated amounts of the single or multivalent compound and infected with X4- or R5-tropic HIV-1. Three days post-infection, a β-galactosidase assay was performed. IC50 values are given in the SI (Table S2). (B) Human PBMCs were isolated, stimulated, and pretreated with 1 μM of the indicated single- or multivalent compound, Maraviroc (MVC/50 nM) or AMD3100 (1 μM). The cells were infected with X4- or R5-tropic HIV-1. Infectious virus yield was determined by infection of TZM-bl reporter cells with PBMC culture supernatants obtained at the indicated day post-infection (dpi). Each curve indicates three biological replicates ± SEM. **p <0.01, ***p <0.001 (one-way ANOVA with reference to SAv).

Next, we investigated the antiviral activity of the multifunctional protein constructs SAv-VIR-102C9 (**12**), SAv-EPI-X4 JM#173-C (**13**), and SAv-VIR-102C9-EPI-X4 JM#173-C (**14**), in vitro. To confirm the sustained antiviral activity of the mono- and multivalent biotin- (B) and SAv-coupled peptides, we conducted HIV-1 infection assays with TZM-bl reporter cells, derived from a HeLa cell clone engineered to stably express CD4, CCR5, and CXCR4.^37^ As a result, TZM-bl cells are highly susceptible to HIV-1 infection and commonly used for studies on viral entry, tropism, neutralization, and drug sensitivity.^38^ TZM-bl reporter cells were pretreated with increasing concentrations of the mono- and multivalent compounds and subsequently infected with the well-characterized X4-tropic HIV-1 NL4-3 molecular clone or an R5-tropic derivative thereof that differs in the V3 region of the viral envelope glycoprotein from the parental virus (Figure 5A).^39^ B-VIR-102C9 (**5**) inhibited both X4- and R5-tropic HIV-1 NL4-3 constructs with mean 50% inhibitory concentrations (IC50) of ∼1.1 and ∼1.2 μM, respectively. The multivalent SAv-VIR-102C9 construct (**12**) showed 11- and 18-fold enhanced antiviral activity (IC50 values of ∼25 and 100 nM or ∼17 and 68 nM per construct or VIR-102C9 content, respectively) compared to the single peptide **5** against X4- and R5-tropic HIV-1 NL4-3. As expected, B-EPI-X4 JM#173-C (**6**) inhibited X4-tropic HIV-1 NL4-3 (IC50: ∼1 μM) but was inactive against the R5-tropic derivative. SAv-EPI-X4 JM#173-C (**13**) inhibited X4-tropic HIV-1 NL4-3 with an IC50 of 0.73 μM per construct and 2.92 μM per peptide. In this case, the multivalent construct did not show enhanced antiviral potency compared to the monomeric peptide **6**. Finally, **14** containing both inhibitory peptides (SAv-VIR-102C9-EPI-X4 JM#173-C) efficiently inhibited both X4- (IC50: 26 nM per construct; 104 nM per bivalent peptide) and R5-tropic (IC50: 39 nM per construct; 156 nM per peptide) HIV-1 NL4-3 infection. Construct **14** showed ∼11- and 8-fold increased inhibitory activity against X4- and R5-tropic HIV-1, compared to monomeric VIR-102C9 (**5**). Taken together, our results support a clear multivalency effect in constructs containing VIR-102C9 (**12, 14**), possibly because the HIV-1 envelope glycoprotein is a trimer and targeting of several gp41 fusion peptides might be required for effective inhibition. Notably, none of the compounds were cytotoxic at the used concentrations (SI Figure S15).

To examine the efficiency of the mono- and multivalent SAv-coupled compounds in inhibiting spreading HIV-1 infection in primary viral target cells, we infected activated peripheral blood mononuclear cells (PBMCs) from three human donors in the presence and absence of the compounds. Infectious virus production was determined by infection of TZM-bl indicator cells with PBMC culture supernatants obtained at different days post-infection (dpi). Predictably, AMD3100, a CXCR4 antagonist clinically approved for mobilizing hematopoietic stem cells,^40^ blocked X4-tropic HIV-1, while the CCR5-antagonist Maraviroc (MVC) prevented R5-tropic HIV-1 replication. All three multivalent constructs (**12**, **13**, and **14)** significantly reduced the replication of X4-tropic HIV-1. VIR-109C2 containing assemblies (**12**, **14**) also reduced the replication of R5-tropic HIV-1 although less efficiently than MVC (Figure 5B). Altogether, the coupled peptides maintained their activity against HIV-1 in primary human cells.

## Conclusions

In this work, we present the synthesis and supramolecular assembly to prepare peptide bispecifics targeting the HIV-1 gp41 fusion peptide or the CXCR4 co-receptor as a proof-of-concept approach for the combination of antiviral peptides acting by different mechanisms on tetrameric SAv. We were able to link three different thiol-reactive moieties in one system using a bis-sulfone moiety in combination with a maleimide functionality. Our procedure offers chemoselectivity by a simple pH control. Notably, we were able to combine two peptide sequences through an internal amino acid modification, which cannot be easily accomplished by standard solid-phase peptide synthesis. With this, therapeutic peptides can be conjugated by adding a natural amino acid side chain and functionalized with an affinity group (biotin), for assembly to form tetravalent bispecifics on a protein nanoplatform. We confirmed the inhibitory effects of the tetravalent SAv-peptide constructs against R5- and X4-tropic HIV-1 variants. Remarkably, the tetravalent SAv-VIR-102C9 and SAv-VIR-102C9-EPI-X4 JM#173-C showed increased inhibitory activity against both X4- (11-fold) and R5-tropic (8-fold) HIV-1, compared to B-VIR-102C9. Our results further revealed that the bispecific tetravalent construct **14** shows increased activity against both X4- and R5-tropic HIV-1 variants. The approach presented herein is not limited to VIR-102C9, EPI-X4 JM#173-C, and Bt-SH but can be used as a versatile platform for the conjugation of any thiol-containing peptides or targeting units. The chemical strategy and supramolecular platform described here can emerge as a convenient tool for the preparation of multifunctional bispecific peptides for potential antiviral treatments including expansion to peptides that target two different viruses. Besides combining two peptides, VIRIP-derived drugs act by a unique mechanism and can be combined with other antiviral drugs with careful chemical design. Finally, our approach offers perspectives for other diseases, such as targeted cancer therapy by addressing two different target receptors on the cell surface.

## Supporting Information Available

Full experimental procedures and characterization data for new compounds (PDF)
