# Supplementary material for: Peptide Bispecifics Inhibiting HIV-1 Infection by an Orthogonal Chemical and Supramolecular Strategy
Source: Bioconjug Chem. 2023 Sep 4;34(9):1645–52. doi: 10.1021/acs.bioconjchem.3c00314 (PMC10515486; doi:10.1021/acs.bioconjchem.3c00314)
Supplement: Supplementary file 1 — bc3c00314_si_001.pdf [file bc3c00314_si_001.pdf]

Supporting information

Peptide Bispecifics Inhibiting HIV-1 Infection by an Orthogonal Chemical and Supramolecular Strategy

Dominik Schauenburg[1, ‡], Fabian Zech[2, ‡], Astrid Johanna Heck[1], Pascal von Maltitz[2], Mirja Harms[2], Siska Führer[1], Nico Alleva[1], Jan Münch[2], Seah Ling Kuan[1, *], Frank Kirchhoff[2, *], Tanja Weil[1, *]

[1]Max-Planck Institute for Polymer Research, Ackermannweg 10, 55128 Mainz, Germany

[2]Institute of Molecular Virology, Ulm University Medical Center, Meyerhofstr. 1, 89081 Ulm, Germany

1. General information and materials....3
2. Organic Synthesis....4
2.1. Synthesis of tri-functional bis-sulfone-PEG-maleimide (2)....4
2.2. Synthesis of Biotin-PEG-SH (10)....8
3. Peptide Conjugation....10
3.1. Biotin-PEG11-VIR-102C9 (B-VIR-102C9, 5)....10
3.2. Biotin-PEG_{11}- EPI-X4 JM#173-C (B-EPI-X4 JM#173-C, 6)....12
3.4. B-VIR-102C9-EPI-X4 JM#173-C (11)....16
4. Assembly of conjugates onto Streptavidin protein platform....19
4.1. 2-((4'-hydroxyphenyl)-azo) benzoic acid (HABA) – Assay....19
4.4. Assembly of B-VIR-102C9-EPI-X4 JM#173-C (11) on Streptavidin....21
5. Atomic Force Microscopy (AFM)....21
6. Materials and Methods for in vitro Studies....23
6.1. Cell culture and Primary cells....23
6.2 Virus stocks....24
6.3 Cell viability....25
6.4 Replication kinetics in PBMCs....25
6.5 Infectious virus....26

1. General information and materials

Unless otherwise stated, all chemicals were obtained from commercial sources (Merck, Sigma Aldrich, Fluka and Thermo Scientific, Fisher Scientific) and used without further purification. All organic solvents (acetonitrile (CH_3CN), chloroform ($CHCl_3$), dichlormethane (CH_2Cl_2), dimethylformamide (DMF), dimethyl sulfoxide (DMSO), ethyl acetate (EtOAc), methanol (CH_3OH), tetrahydrofuran (THF)) were obtained from Fisher Scientific and used without further purification (HPLC or analytical grades). H_2O used for the reactions was obtained from the Millipore purification system. Reaction progress was monitored by thin layer chromatography (TLC) using silica pre-coated aluminum sheets (0.2 mm Silica with fluorescence indicator UV 254 nm from Marcherey-Nagel). For visualization ultraviolet lamp (254 nm) or potassium permanganate staining solution (3 g $KMnO_4$, 20 g K_2CO_3, 5 mL 5% NaOH and 300 mL H_2O), ninhydrin (1.5 g ninhydrin in 500 mL methanol and 15 mL acetic acid) were used. Flash column chromatography was carried out using Merck silica gel 60 mesh (pore size 60 Å, 230–400 mesh particle size). NMR spectra were recorded on Bruker Avance 300, 500 or 700 MHz NMR spectrometer in the stated solvents (d_6-DMSO, $CDCl_3$, CD_3CN, D_2O, CD_3OD). Chemical shifts (δ) were reported as parts per million (ppm) referenced with respect to the residual solvent peaks. Multiplicity was descripted as followed: s = singlet, d = doublet, t = triplet, dd = doublet of doublets, dt = doublet of triplets, m = multiplet, br = broad. Liquid chromatography-mass spectroscopy (LC-MS) analysis was performed on a Shimadzu LC-MS 2020 equipped with an electrospray ionization source, a SPD-20A UV-Vis detector and a Kinetex EVO C18 column (2.1 × 50 mm, 2.6 μm). UV-traces are presented with subtracted blank. Maldi-ToF spectra were acquired on a Bruker Time-of-flight MS rapifleX MALDI-ToF-MS equipped with a 10 kHz scanning smartbeam 3D laser (Nd:YAG at 355 nm) and a 10 bit 5 GHz digitizer. HR-ESI-MS was recorded using WATERS SYNAPT G2-Si mass spectrometer. The absorbance was measured on a microplate reader (Tecan Spark 20M) using a Greiner 384 well UV-Star microplate or a nanodrop 1000 Spectrophotometer.

2. Organic Synthesis

2.1. Synthesis of tri-functional bis-sulfone-PEG-maleimide (2)

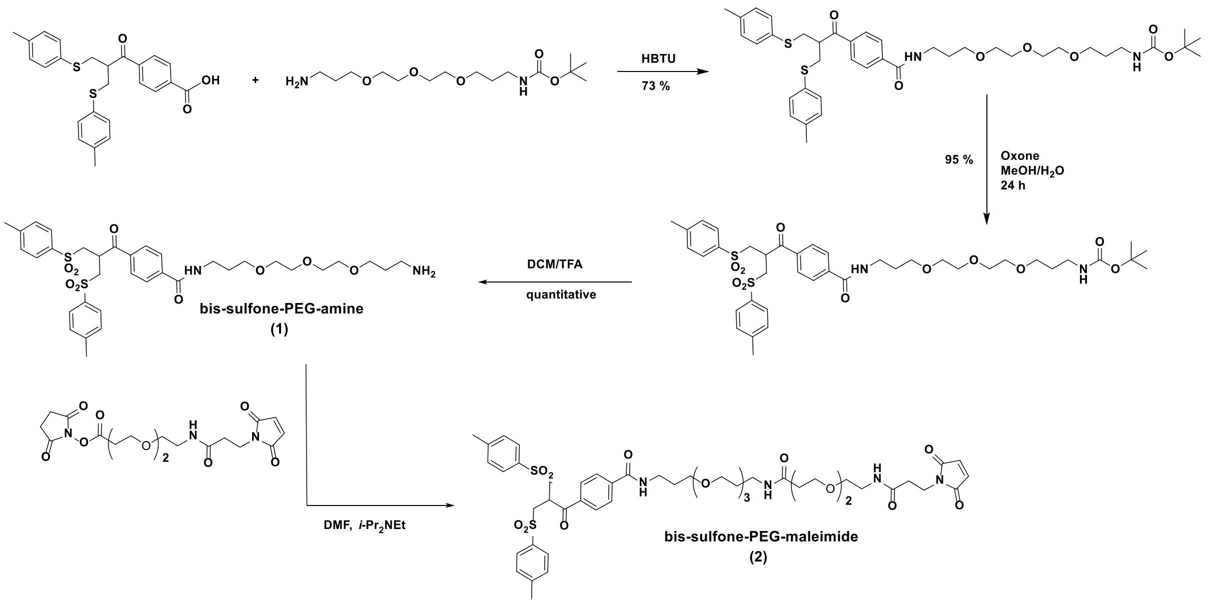

Scheme 1: Synthesis of bis-sulfone-PEG-amine as previously reported with modifications (top).[1]Synthesis route of bis-sulfone-PEG-maleimide (**2**, bottom).

***N*-(27-(2,5-dioxo-2,5-dihydro-1*H*-pyrrol-1-yl)-15,25-dioxo-4,7,10,17,20-pentaoxa-14,24-diazaheptacosyl)-4-(3-tosyl-2-(tosylmethyl)propanoyl)benzamide (bis-sulfone-PEG-maleimide, 2)**

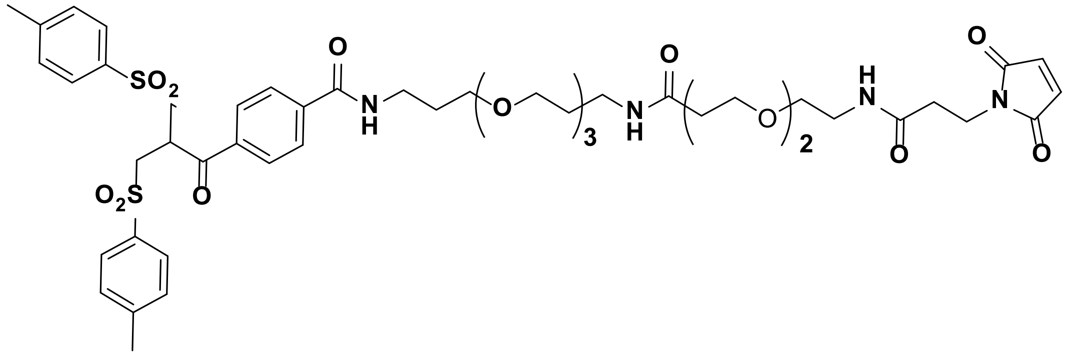

N-(3-(2-(2-(3-aminopropoxy)ethoxy)ethoxy)propyl)-4-(3-tosyl-2 (tosylmethyl)propanoyl)benzamide TFA salt (bis-sulfone-PEG-amine, **1**)[1] (100 mg, 142 µM, 1.0 equiv) was dissolved in anhydrous DMF (15 mL). To this solution *i*-Pr_2NEt (24.7 µl, 142 µM, 1.0 equiv) was added. In a separate vial commercially available 3-[2-[2-[[3-(2,5-Dihydro-2,5-dioxo-1H-pyrrol-1-yl)-1-oxopropyl]amino]ethoxy]ethoxy]propanoic acid

[1] Wang, Tao, et al. "A Disulfide Intercalator Toolbox for the Site- Directed Modification of Polypeptides." *Chemistry–A European Journal* 21.1 (2015): 228-238.

2,5-dioxo-1-pyrrolidinyl ester (maleimide-PEG$_2$-NHS, 94 mg, 212 µM, 1.5 equiv) was dissolved in 1 mL anhydrous DMF. The solution of the NHS-ester was added dropwise to the bis-sulfone PEG-amine and the mixture was stirred at rt overnight. The next day LC-MS analysis showed full conversion of the bis-sulfone PEG-amine. The solvent was removed under vacuum at rt, the residue was dissolved in CH_3CN/H_2O (1:1, v/v) containing 0.1 % formic acid and purified by preparative HPLC. ((HPLC gradient: 10% B for 4 min, 95% B in 20 min, 95% B for 3 min.)

Product containing fractions were collected as a mixture of maleimide bis-sulfone (**2**) and maleimide allyl-sulfone (**2-eli**) and lyophilized.

Note: During the amide bond coupling partial elimination of the bis-sulfone to the allyl-sulfone was observed. These two compounds can be separated by preparative HPLC however, separation at this step is not necessary since the first Michael addition to the maleimide is selective under weekly acidic conditions (pH 6.0).

Yield:(28 mg, 27.6 µmol, 39 %)

Chemical formula: $C_{49}H_{64}N_4O_{15}S_2$

LC-MS (ESI): Tr = 6.4 min, m/z = 1013.5 $[M+H]^+$ (calc. 1013.4). 1035.5 $[M+Na]^+$ (calc. 1036.4).

HR-ToF-MS (ESI): m/z = 1013.3881 $[M+H]^+$ (calc. 1013.3882).

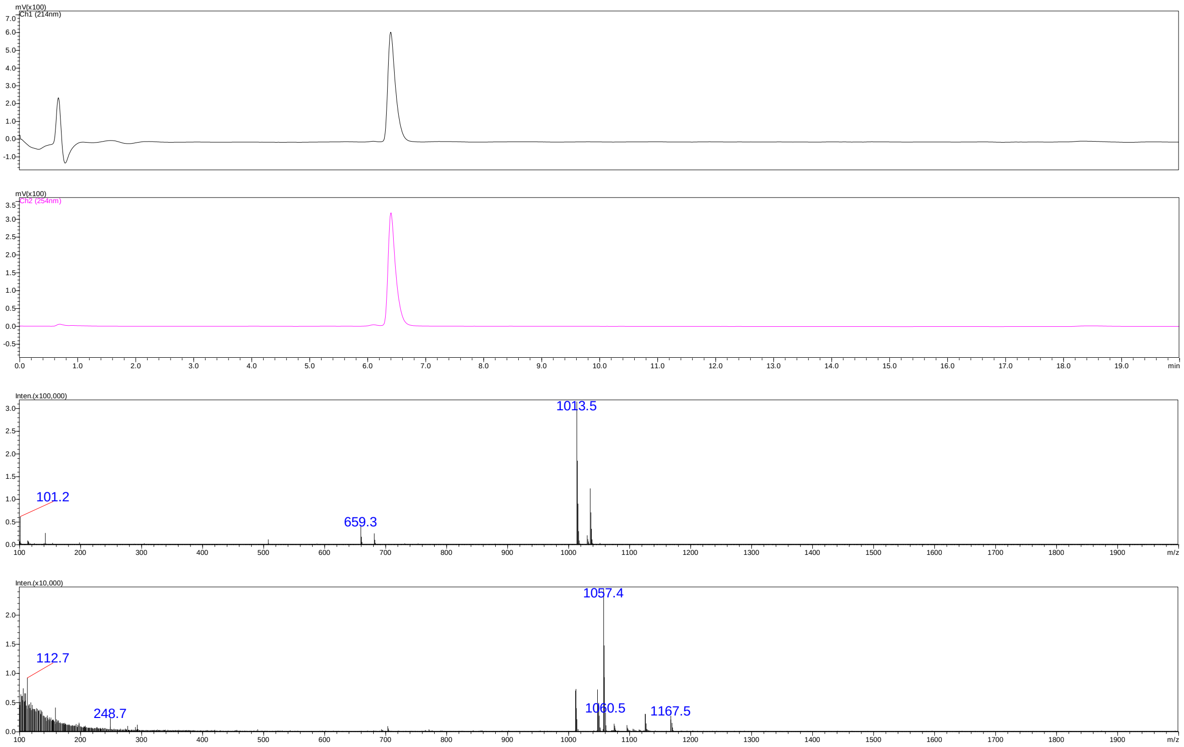

Figure S1: LC-MS spectrum of bis-sulfone PEG-maleimide, **2** (Tr = 6.4 min) at 214 nm (top) and 254 nm (bellow), B: LC-MS spectrum of bis-sulfone PEG-maleimide, **8** (Tr = 6.4 min) at 254 nm, C: LC-MS

spectrum of spectrum of bis-sulfone PEG-maleimide, **2** positive ionization mode (2nd from bottom) and negative ionization mode (bottom).

(a)

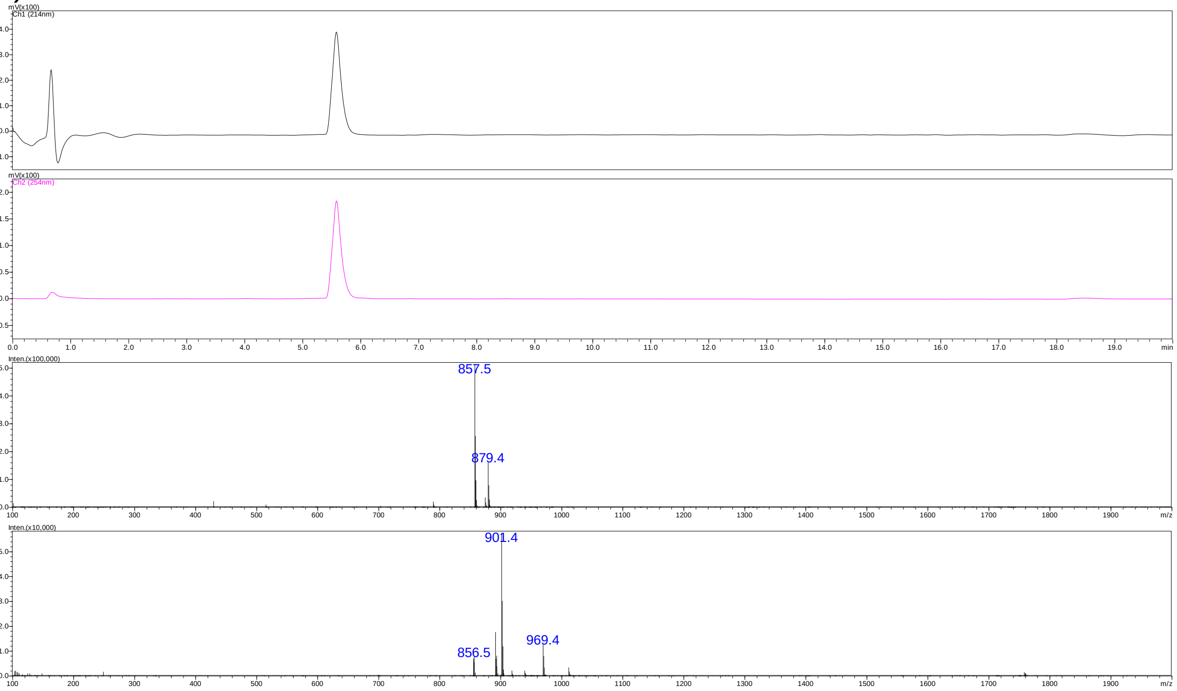

(b)

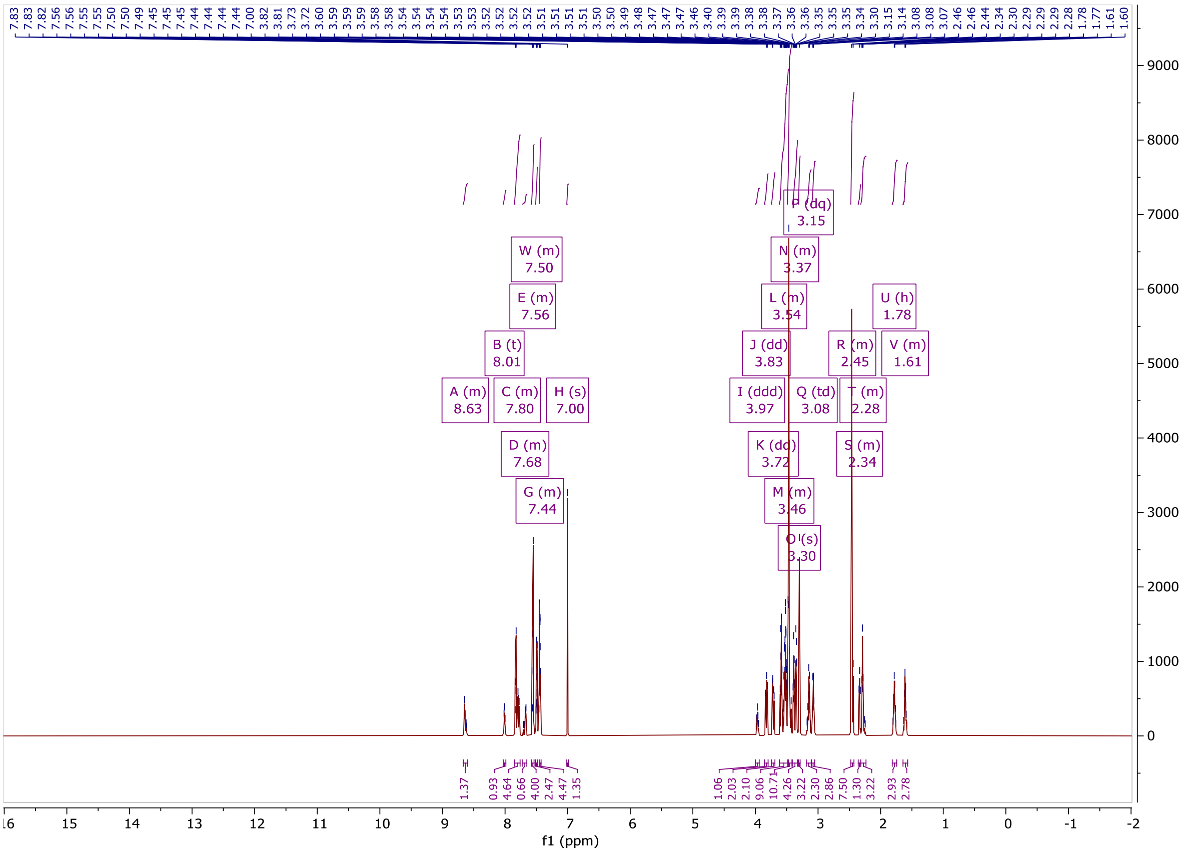

Figure S1: (a) LC-MS spectrum of allyl-sulfone PEG-maleimide **eli-2**, (Tr= 5.6 min) at 214 nm (top); LC-MS spectrum of allyl-sulfone PEG-maleimide **eli-2**, (Tr= 5.6 min) at 254 nm (below); LC-MS spectrum of spectrum of allyl-sulfone PEG-maleimide **eli-2**, positive ionization mode (m/z = 857.5

[M+H]$^+$) (2nd from bottom). LC-MS spectrum of allyl-sulfone PEG-maleimide **eli-2**, negative ionization mode (m/z = 856.5 [M-H]$^-$) (bottom) (b) ^{1}H NMR spectrum

2.2. Synthesis of Biotin-PEG-SH (10)

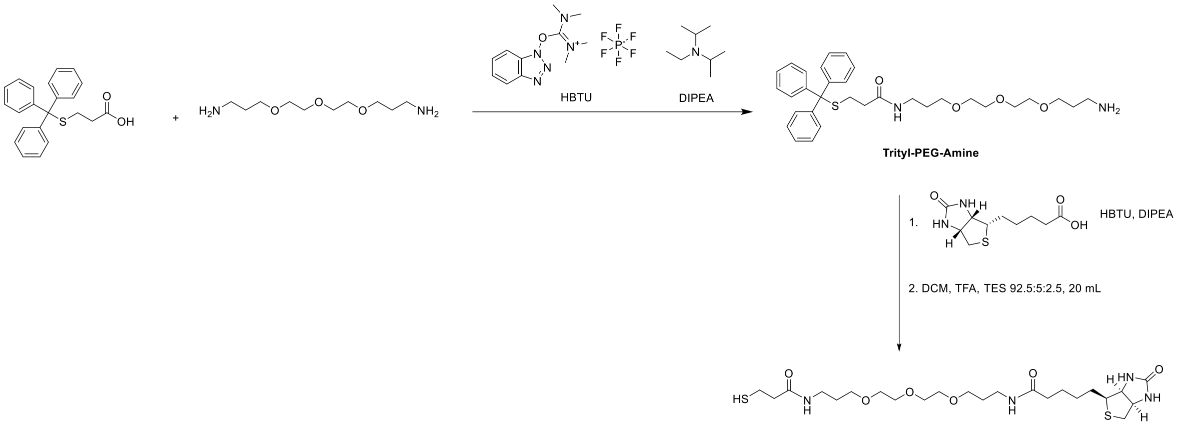

Biotin-PEG-SH (10)

Scheme 2: Synthesis of Biotin-PEG-SH (11) in a three steps synthesis with modifications as previously reported.[2]

*N-(*17-mercapto-15-oxo-4,7,10-trioxa-14-azaheptadecyl)-5-((3aS,4S,6aR)-2-oxohexahydro-1H-thieno[3,4-d]imidazol-4-yl)pentanamide (10)

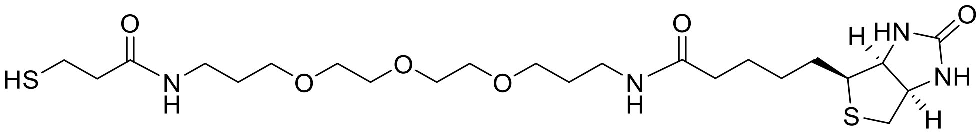

Biotin (266.15 mg, 1.09 mmol, 1.2 equiv.) was dissolved in 2 mL DMSO. HBTU (413.15 mg, 1.09 mmol, 1.2 equiv.) and DIPEA (308,78 µL, 234,67 mg, 1,82 mmol, 2 equiv.) were dissolved in 5 mL anhydrous, peptide grade DMF and added to the Biotin. The mixture was kept stirring for 15 min. Then, N-(3-(2-(2-(3-aminopropoxy)ethoxy)–ethoxy)propyl)-3-(tritylthio)propenamide (trityl-PEG3-amine, 500.0 mg, 907.84 µmol, 1 equiv) in 3 mL mL anhydrous, peptide grade DMF were added to the mixture and kept stirring over night at rt. The solvent was evaporation in vacuo and redissolved in 20 mL DCM. The solution was extracted twice with 20 mL 1M $NHCO_3$. Water phase was extracted twice with 20 mL DCM. The combined organic layers were dried over $MgSO_4$ and solvent was removed in vacuo. For trityl deprotection the crude was dissolved in 40 mL DCM, TFA, TIPS, EDT (90:5:2.5:2.5) and kept stirring for 4h. After solvent removal the crude was redissolved in MilliQ/CH_3CN with 0.1 % TFA and purified preparative HPLC, which yielded 297 mg of product **10.**

---

[2] Weinrich, D., Köhn, M., Jonkheijm, P., Westerlind, U., Dehmelt, L., Engelkamp, H., Christianen, P.C., Kuhlmann, J., Maan, J.C., Nüsse, D. and Schröder, H., 2010. Preparation of biomolecule microstructures and microarrays by thiol–ene photoimmobilization. *ChemBioChem*, *11*(2), pp.235-247.

Yield: 297 mg, 555 µmol, 61%.

Chemical formula: $C_{23}H_{42}N_4O_6S_2$.

LC-MS (ESI): Tr= 3.8 min, m/z = 535.4 $[M+H]^+$ (calc. 535.2619).

^{1}H-NMR ($CDCl_3$, 500 MHz):: δ = 4.59-4.46 (m, 1H, CH-CH2-S), 4.39-4.26 (m, 1H, CH-CH-S), 3.71-3.49 (m, 12H, 6 CH2-O), 3.44-3.26 (m, 4H, 2 CH2-NH), 3.22-3.06 (m, 1H, S-CH-CH), 2.91 (dd, 2 J = 12.9 Hz, 3 J = 4.7 Hz, 1H, S-CHaCH), 2.84-2.70 (m, 3H, S-CHb-CH, CH2-SH), 2.48 (t, 3 J = 6.8 Hz, 2H, CH2-CH2-SH), 2.21 (t, 3 J = 7.4 Hz, 2H, CH2-CH2-CH2-CO-NH), 1.84-1.73 (m, 4H, CO-NH-CH2-CH2), 1.72-1.57 (m, 4H, CH2-CH2-CH2-CO-NH, CH2-CH2-CH2-CO-NH), 1.50-1.35 (m, 2H, S-CH-CH2).

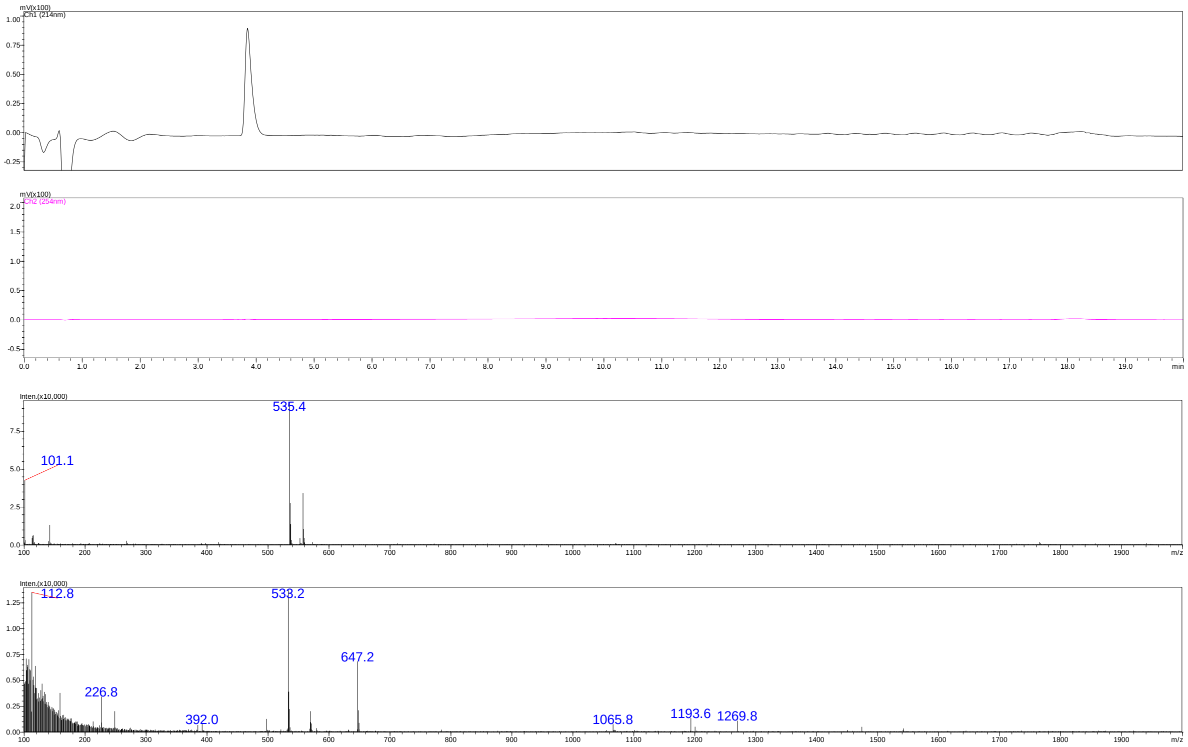

Figure S2: LC-MS spectrum of Biotin-PEG-SH (**10**) at 214 nm (top) and 254 nm (below) Tr = 3.8 min. ESI spectrum, positive ionization mode (2nd from bottom) negative ionization mode (bottom).

3. Peptide Conjugation

3.1. Biotin-PEG11-VIR-102C9 (B-VIR-102C9, 5)

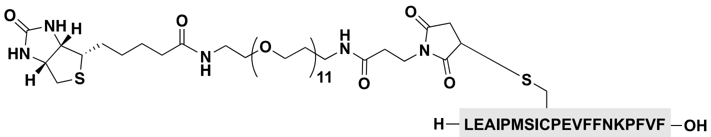

The peptide VIR-102C9 (**3**, 5.0 mg, 2.1 µmol, 1 equiv.) was dissolved in 5 mL of sodium phosphate (PB) buffer (50 mM, pH 6.8) containing 20 % CH_3CN (v/v). The reaction solution was heated to 40°C, to fully dissolve the peptide. Tris(2-carboxyethyl)phosphine) (TCEP, 1 mg/mL in PB, 537 µg, 1 equiv.) was added and the mixture stirred at 40°C for 1 h. Commercially available maleimide-PEG$_{11}$-biotin (Thermo Fisher Scientific, Waltham, Massachusetts, USA, 2.88 mg, 3.1 µmol, 1.5 equiv.) was dissolved in 288 µL DMF and added to the peptide solution. The mixture stirred at 40°C overnight. Solvents were removed through lyophilization and the crude product was redissolved in 2 mL MilliQ with 20 % CH_3CN (v/v) and 0.1 % TFA. Purification was achieved by semi preparative HPLC using an Eclipse XDB-C18 column (1.4 x 250 mm, 5 µm, Agilent) under acidic conditions. Mobile Phase 0.1% TFA in MilliQ was used as solvent A, and 0.1%TFA in CH_3CN was used as solvent B. (HPLC gradient: 5% B for 3 min, 95% B in 20 min, 95% B for 2 min.)

The product containing fractions were freeze dried yielding in 4.3 mg of compound **5**.

Yield: 4.3 mg, 1.32 µmol, 62 %.

Chemical Formula: $C_{154}H_{237}N_{27}O_{43}S_3$.

LC-MS (ESI): Tr = 5.4 min, m/z = 813.7 $[M+4H]^{4+}$, 1084.5 $[M+3H]^{3+}$ (calc. 1083.9), 1626.6 $[M+2H]^{2+}$ (calc. 1625.3).

ToF-MS (ESI): m/z = 813.1458 $[M+4H]^{4+}$ (calc. 813.1660), 1083.8579 $[M+3H]^{3+}$ (calc. 1083.8856), 1625.2816 $[M+2H]^{2+}$ (calc. 1625.3248).

ToF-MS (MALDI), CHCA: m/z = 3249.5168 $[M+H]^+$ (calc. 3248.6351), 3271.4811 $[M+Na]^+$ (calc. 3271.6243), 3287.4507 $[M+K]^+$ (calc. 3287.5982).

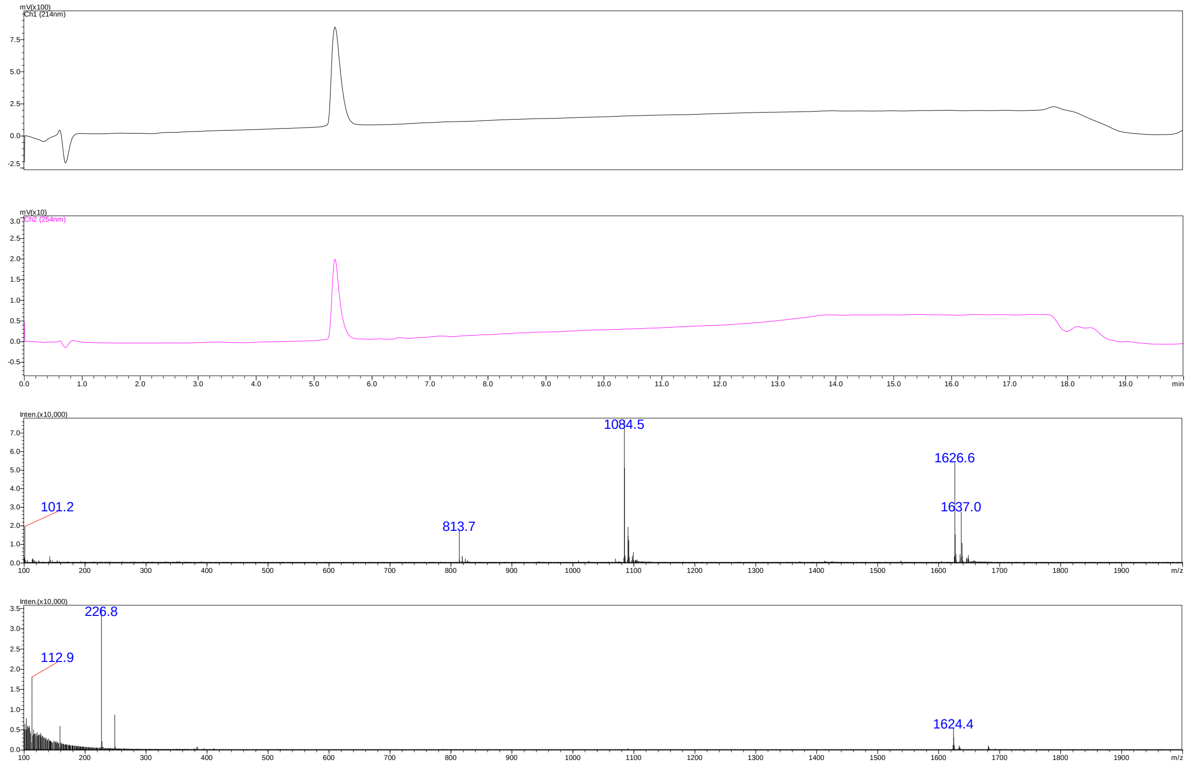

Figure S3: LC-MS spectrum of B-VIR-102C9, **5:** UV-trace at 214 nm (top) and 254 nm (Tr = 5.4 min) (bellow); ESI spectrum positive ionization mode m/z (calc.) = 3248.6 [M], m/z (found) = 1626.6 $[M+2H]^{2+}$, 1084.5 $[M+3H]^{3+}$) (2nd from bottom); ESI spectrum negative ionization mode(bottom) m/z (found) = 1624.4 $[M-2H]^{2-}$ (bottom)

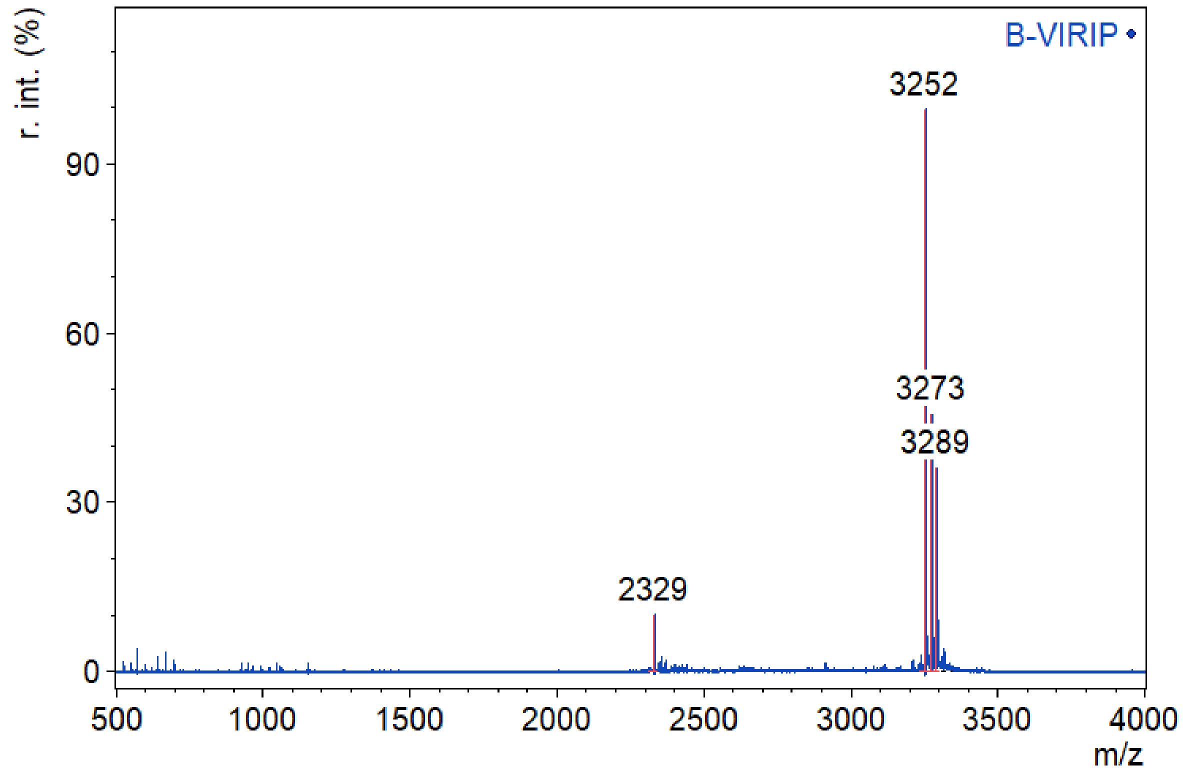

Figure S4: MALDI-ToF mass spectrum of the isolated compound ***5***. Measurement was performed using a alpha-Cyano-4-hydroxycinnamic acid (CHCA), presented as average mass. m/z = 2329 $[\mathbf{2}+H]^+$, 3252 $[M+H]^+$, m/z = 3273 $[M+Na]^+$, 3289 $[M+K]^+$

3.2. Biotin-PEG11- EPI-X4 JM#173-C (B-EPI-X4 JM#173-C, 6)

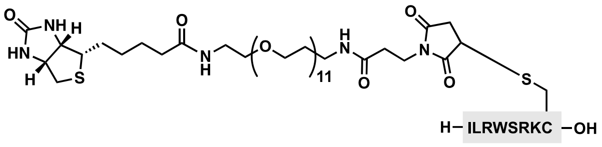

The peptide EPI-X4 JM#173-C (**4**, 5.0 mg, 4.72 µmol, 1 equiv.) was dissolved in 5 mL of sodium phosphate (PB) buffer (50 mM, pH 6.8) and Tris(2-carboxyethyl)phosphine) (TCEP, 1 mg/mL in PB, 537 µg, 1 equiv.) was added and stirred for 1 h. Commercially available maleimide-PEG$_{11}$-biotin (Thermo Fisher Scientific, Waltham, Massachusetts, USA, 2.88 mg, 3.1 µmol, 1.5 equiv.) was dissolved in 288 µL DMF and added to the peptide solution. The mixture stirred at rt overnight. Solvents were removed through lyophilization, and the crude product was redissolved in 2 mL MilliQ with 0.1 % TFA. Purification was achieved by semi preparative HPLC using an Eclipse XDB-C18 column (1.4 x 250 mm, 5 µm, Agilent) under acidic conditions. Mobile Phase 0.1% TFA in MilliQ was used as solvent A, and 0.1%TFA in CH_3CN was used as solvent B. (HPLC gradient: 5% B for 3 min, 95% B in 20 min, 95% B for 2 min.)

The product containing fractions were lyophilized and yielded 6.3 mg (67%) of compound **6**.

Yield: 6.3 mg, 3.18 µmol, 67 %.

Chemical formula: $C_{88}H_{152}N_{22}O_{25}S_2$.

ToF-MS (MALDI), CHCA : m/z = 1983 $[M+H]^+$ (calc.1982).

(a)

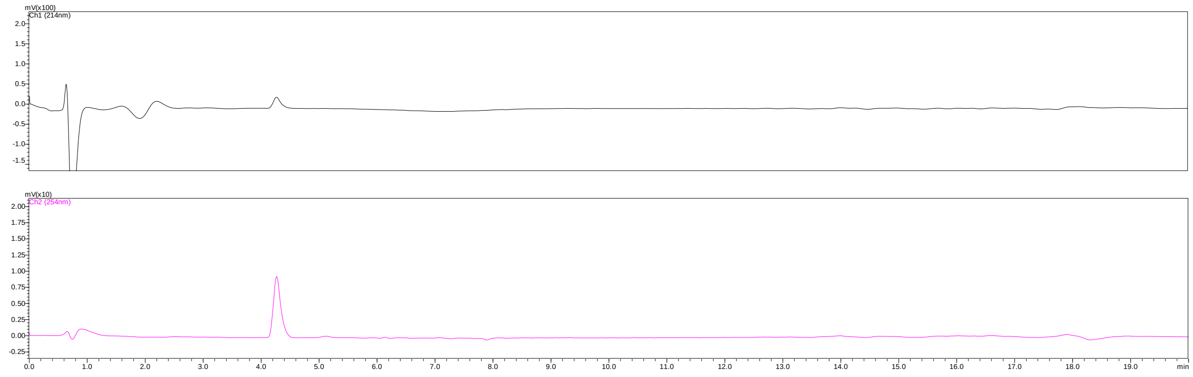

(b)

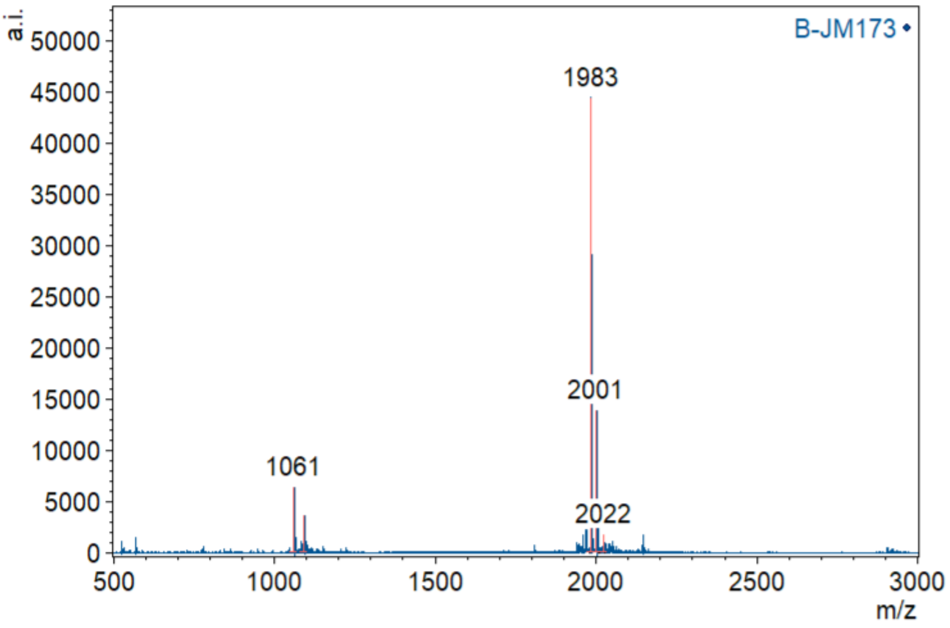

Figure S5: (a) Liquid chromatogram showing purity 214 nm (top) and 254 nm (bottom); (b) MALDI-ToF mass spectrum of the isolated compound **6**. Measurement was performed using a CHCA matrix, presented as average mass. m/z = 1983 [M+H$^+$], sodium adduct m/z = 2001 [M+Na$^+$].

3.3. VIR-102C9-bis-sulfone (7)

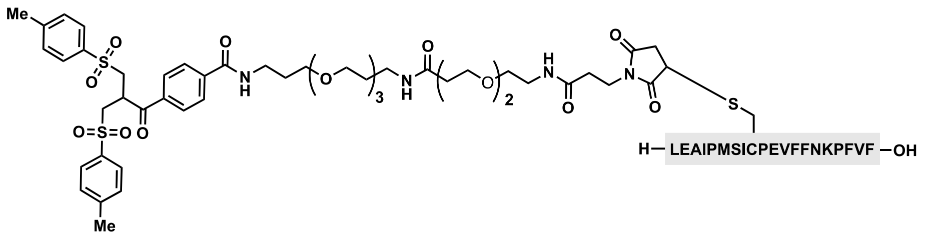

The peptide VIR-102C9 (**3**, 9.58 mg, 4.11 µmol, 1.0 equiv) and TCEP (1.03 mg, 4.11 µmol, 1.0 equiv) were dissolved in 5 ml of sodium phosphate (PB) buffer (50 mmol PB, pH 6.0, containing 40% acetonitrile, v/v) and stirred for 30 min at rt. Bis-sulfone-PEG-maleimide, (**2**, 5 mg, 4.11 µmol, 1.0 equiv) was dissolved in 3 ml PB buffer (50 mmol, pH 6.0, containing 40% acetonitrile, v/v). The reaction mixture was stirred for 2 h at rt, and purified by preparative HPLC (HPLC gradient: 5% B for 3 min, 95% B in 20 min, 95% B for 2 min.) Product containing fractions were collected as a mixture of VIR-102C9 bis-sulfone (**7**) and VIR-102C9 allyl-sulfone (**8**) and lyophilized

Note: At this step VIR-102C9 bis-sulfone and VIR-102C9 allyl-sulfone can be separated by preparative HPLC. Since the next step of reaction course is the elimination of the bis-sulfone to the allyl-sulfone under slight basic conditions (pH 8.0), no separation is required. For analytical purpose of the bis-sulfone the two species were separated.

Yield: 6.59 mg, 1.64 µmol, 40 %.

Chemical formula: $C_{162}H_{230}N_{26}O_{42}S_4$.

LC-MS (ESI) VIR-102C9 bis-sulfone: Tr = 6.6 min, m/z = 1115.0 $[M+3H]^{3+}$ (calc. 1114.0), 1672 $[M+2H]^{2+}$ (calc.1670.7850).

ToF-MS (ESI): m/z = 1670.7899 $[M+2H]^{2+}$ (calc. 1670.7850).

ToF-MS (MALDI), CHCA : m/z = 3339.8866 $[M+H]^{+}$ (calc. 3340.5617), 3361.2211 $[M+Na]^{+}$ (calc. 3362.5436), 3382.5199 $[M+ACN+H]^{+}$ (calc. 3381.8552).

LC-MS (ESI) VIR-102C9 allyl-sulfone: Tr = 6.1 min, m/z = 1593 $[M+2H]^{2+}$ (calc. 1592.7722), 1062.00 $[M+3H]^{3+}$ (calc. 1062.1839), 1592 $[M-2H]^{2-}$ (calc. 1590.7577).

ToF-MS (ESI):m/z = 1592.7870 (calc.1592.7722)

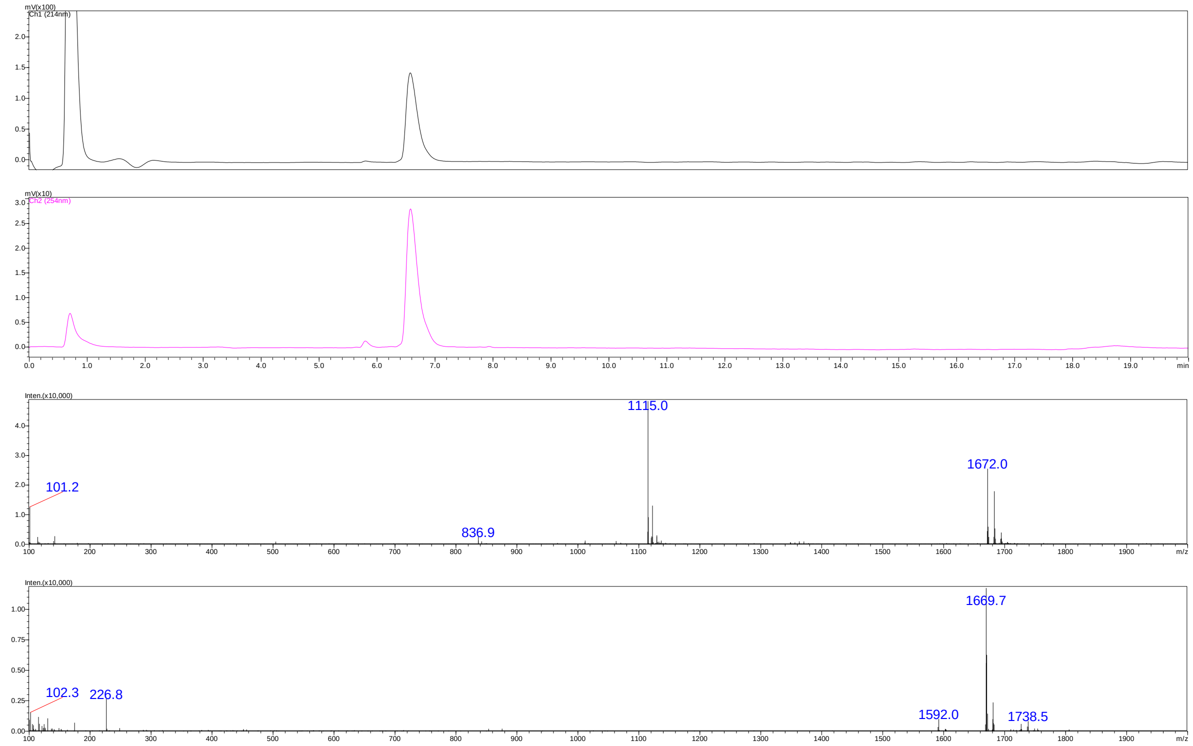

Figure S6: A: LC-MS spectrum of VIR-102C9 bis-sulfone, **7** (Tr = 6.6 min) at 214 nm (top); LC-MS spectrum of VIR-102C9 bis-sulfone **7**, (Tr = 6.6 min) at 254 nm (bellow); ESI spectrum of spectrum of VIR-102C9 bis-sulfone **7**, positive ionization mode (m/z (calc.) = 3339.6 [M], m/z (found) = $[M+2H]^{2+}$ = 1671.0 Da, $[M+3H]^{3+}$ = 1114.0 Da) (2nd from bottom); LC-MS spectrum of VIR-102C9 bis-sulfone **7**, negative ionization mode (m/z (calc.) = 3339.6 [M], m/z (found) = 1669.7 $[M-2H]^{2-}$ (bottom).

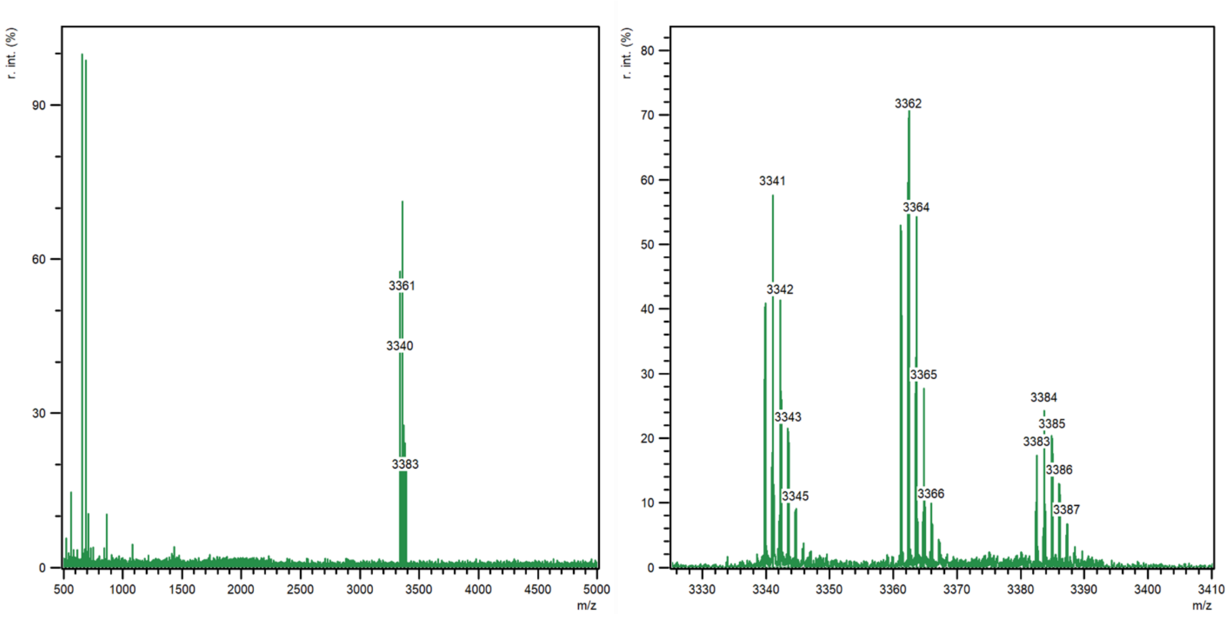

Figure S7: MALDI-ToF mass spectrum of the isolated compound **7** using CHCA matrix,presented as average mass. Full spectrum left, zoom in right. m/z = 3340 $[M+H]^{+}$.

3.4. B-VIR-102C9-EPI-X4 JM#173-C (11)

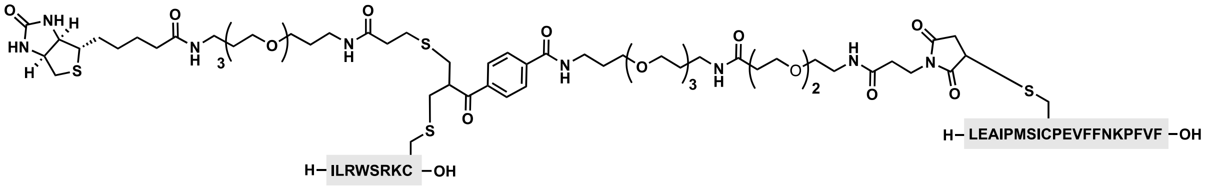

VIR-102C9 bis-sulfone **7** (5 mg, 1.5 µmol, 1.0 equiv) was dissolved in 5 ml of 50 mM PB, 10 mM EDTA, pH 8 and incubated at rt for 24 h. Elimination of the bis-sulfone to the allyl-sulfone can be monitored by LC-MS. EPI-X4 JM#173-C **3** (1.6 mg, 1.5 µmol, 1.0 equiv) was dissolved in 1 ml, 10 mM PB, 2 mM EDTA, pH 8 and TCEP (374 µg, 1.5 µM, 1.0 equiv) was added and gently agitated at rt for 30 min. The EPI-X4 JM#173-C solution was added to VIR-102C9 and shaken for 1 h at rt. Reaction was monitored using LC-MS until EPI-X4 JM#173-C peak disappeared. Biotin-PEG$_3$-SH **10** (8 mg, 15 µmol, 10.0 equiv) was pre-dissolved in 800 µL DMSO and added to 3.2 ml 10 mM PB, 2 mM EDTA, pH 8. TCEP (3.74 mg, 15 µmol, 10.0 equiv) was added and the mixture was kept stirring at rt for 30 min. Then Bt-PEG$_3$-SH solution was added to the B-VIR-102C9-EPI-X4 JM#173-C mixture and kept stirring over night at rt. The resulting mixture was purified by semi-prep HPLC (HPLC gradient: 5% B for 3 min, 95% B in 20 min, 95% B for 2 min). The product containing fractions were lyophilized to obtain 950 µg with an overall yield of 14 %.

As we observed side reactions caused by thiol exchange at higher pH that gave inconsistent yield, subsequent batches were performed using the following protocol:

VIR-102C9 bis-sulfone **7** (500 µg, 0.15 µmol, 1.0 equiv) was dissolved in 500 µL of 50 mM PB pH 8.0 and incubated at rt for 20 h under shaking. The mixture was diluted with 2 mL 50mM PB buffer pH 7.4 to decrease the pH. Then EPI-X4 JM#173-C **3** (0.16 mg, 0.15 µmol, 1.0 equiv) was dissolved in 160 µL MilliQ and added to the VIR-102C9 solution. The mixture was incubated under shaking for 1 h at 25°C. Biotin-PEG3-SH **10** (0.8 mg, 1.5 µmol, 10.0 equiv) was dissolved in 800 µL MilliQ and added to the B-VIR-102C9-EPI-X4 JM#173-C mixture and kept stirring for 14h at 25°C. The resulting mixture was purified by semi-prep HPLC. The product containing fractions were lyophilized 80 µg (12 % yield).

Chemical formula: $C_{218}H_{337}N_{47}O_{53}S_5$.

HR-ToF-MS (ESI): m/z = 925.2822 $[M+4H]^+$ (calc. 925.2823).

ToF-MS (MALDI), CHCA : m/z = 2311.6811 $[M+2H]^+$ (calc. 2311.6934), 4622.4349 $[M+H]^+$(calc. 4622.3796).

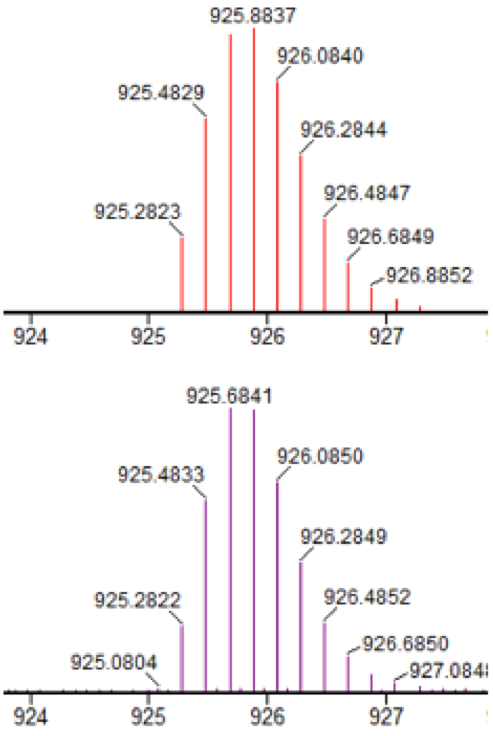

Figure S8: HR-ESI mass spectrum of the isolated compound **11.** Top: calculated isotopic pattern. Bottom: observed isotopic pattern.

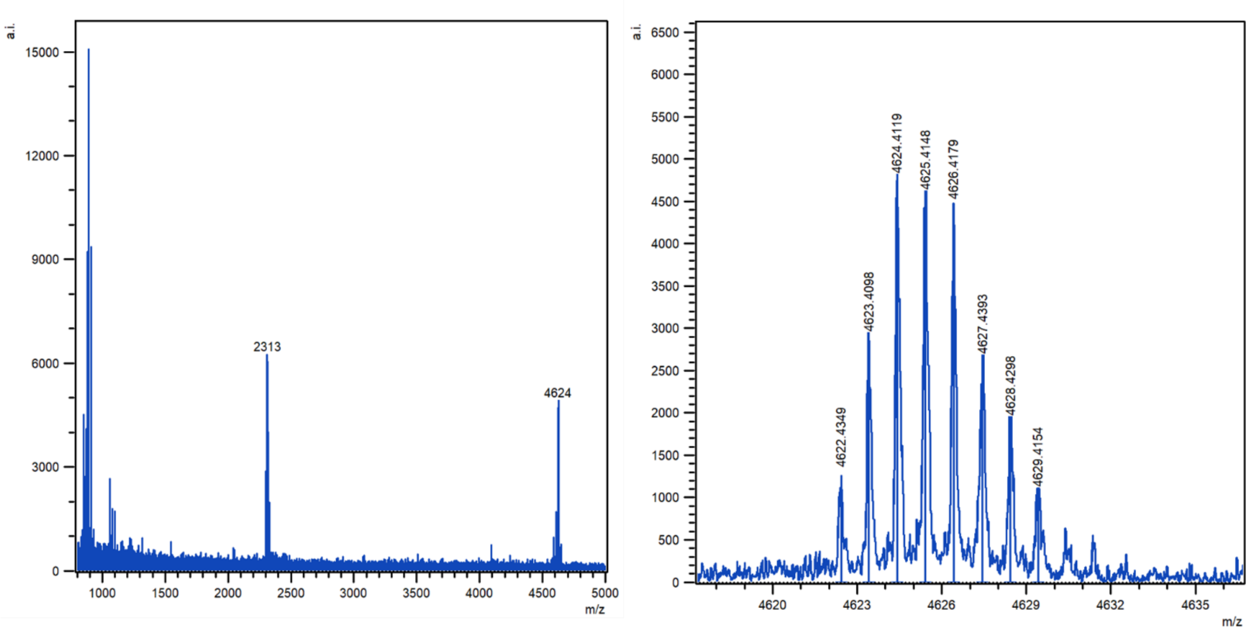

Figure S9: MALDI-ToF mass spectrum of the isolated compound **11.** left: full spectrum showing average mass. right: zoom on isotopic pattern of $[M+H]^+$.

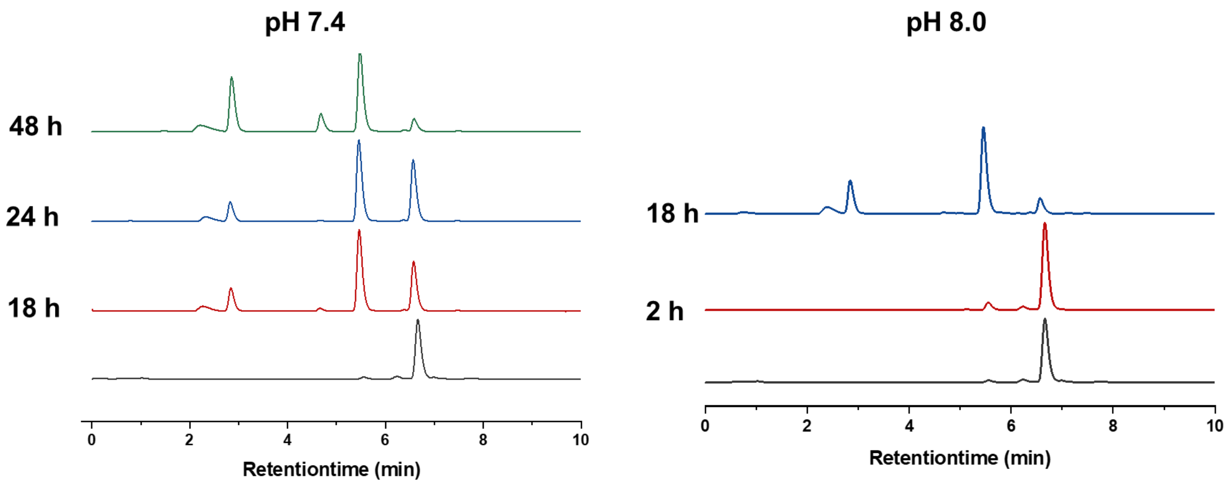

Figure S10: Monitoring of elimination process of bis-sulfone to the allyl-sulfone at pH 7.4 (left) and pH8 (right).

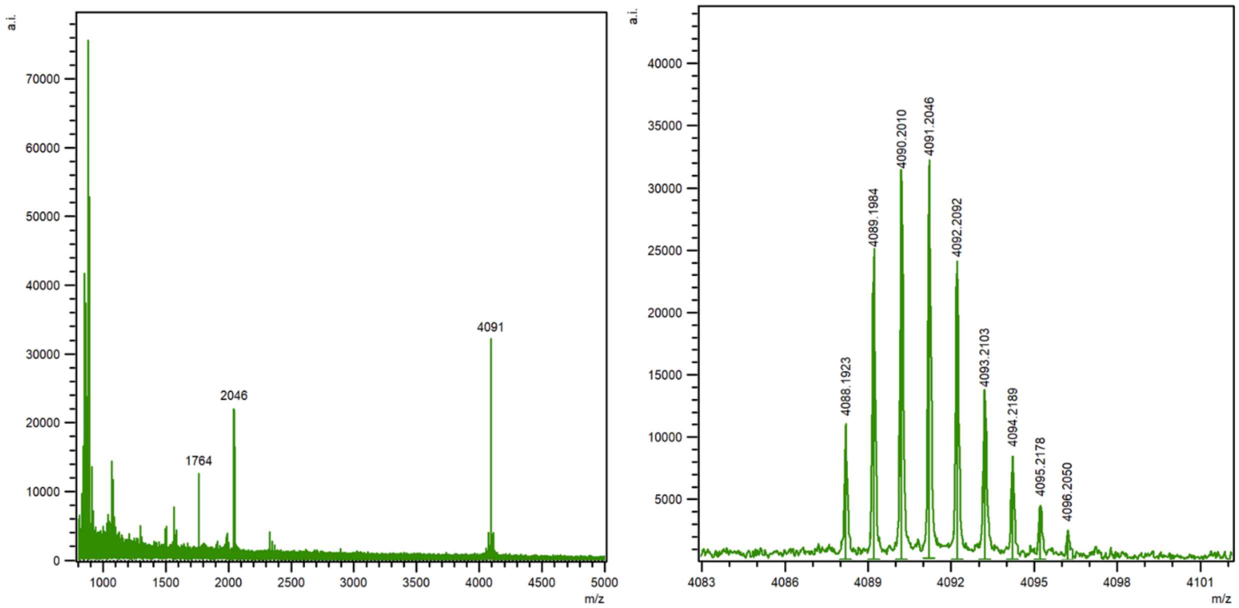

Figure S11: MALDI-ToF mass spectrum of the isolated intermediate **9.** left: full spectrum showing average mass. right: zoom on isotopic pattern of $[M+H]^+$.

4. Assembly of conjugates onto Streptavidin protein platform

4.1. 2-((4'-hydroxyphenyl)-azo) benzoic acid (HABA) – Assay

To determine the required equivalents of biotinylated peptides for saturation of the biotin binding pockets of Streptavidin (SAv, Agilent Technologies), 2-((4'-hydroxyphenyl)-azo) benzoic acid (HABA) was used. Therefore, biotin binding to SAv was analyzed by using seven vials with each 28 µL of a SAv solution (2.5 mg/mL, 1.3 nmol, 1 equiv), in phosphate buffer (50 mM, pH 7.4). Different equivalents of biotin and B-peptides (0 to six equiv. 3.18 µL, .3 nmol, 1equiv; 8.36 µL, 2.6 nmol, 2 equiv and so on) were added to each vial than buffer was added to obtain a total volume of 70 µL. For B-VIRIP-EPI-X4 JM#173-C, up to five equiv was applied. The vials were vortex, incubated for 15 min and spun down to allow complex formation. Triplets of each sample (25 µL) were introduced to a flat-bottomed transparent 384-well plate (UV-star®, Greiner Bio-one GmbH, Frickenhausen, Germany). UV-VIS absorbance spectrum was measured from 250 nm to 850 nm by using a Tecan Spark 20M microplate reader (Tecan Trading AG, Männedorf, Switzerland). The absorption at 500 nm was plotted against the biotin equivalences per protein to give the stoichiometric ratio required for saturation of all binding pockets.

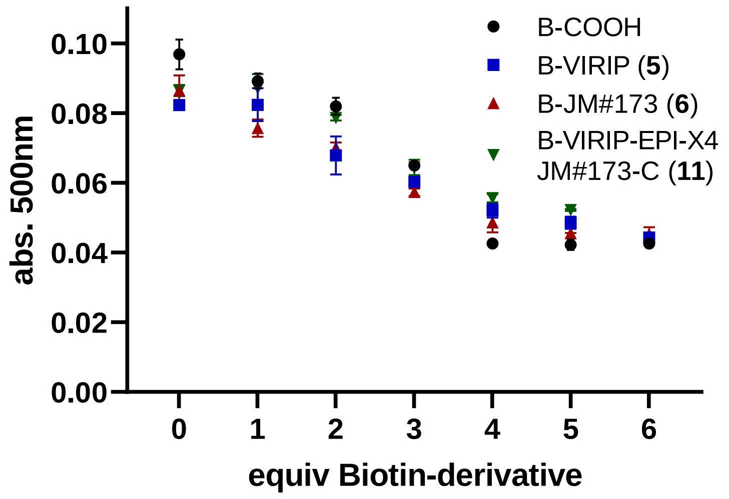

Figure S12: HABA-Assay of Biotin and Streptavidin. All peptides show saturation after 4 mol equiv. per protein (for B-VIRIP-EPI-X4 JM#173-C (**11**) assembly was performed with up to 5 equiv).

4.1.2 Atomic Force Microscopy (AFM)

30 µL of a 10 µg mL^{-1} protein solution (MilliQ H_2O) was dropped on a freshly cleaved mica plate and absorbed for 10 min at room temperature. After addition of 70 µL MilliQ H_2O samples were imaged using liquid tapping mode on a Bruker Dimension FastScan Bio AFM instrument equipped with the ScanAsyst mode with scan rates of 1.4 Hz. Images were analyzed by using NanoScope Analysis 1.8 software.

4.1.3 SDS-PAGE analysis

Preparation of samples for SDS-PAGE follows standard protocol by Bio-Rad on a Mini-Protean TGX 4-20% pre casted gel (Bio-Rad Laboratories, USA), using Laemmli protein sample buffer, with and without 10 vol% ethantiol (sample heating to 95 °C for 5 min) as denaturation and reduction steps. The gel is run in Tris/Glycine/SDS buffer (Bio-Rad Laboratories, USA) with a constant 140 V for 60 min using EXtended PS 13 (5 - 245 kDa) ladder as the reference. Samples were loaded in the gel from left to right: ladder, SAv, SAv (reducing, boiled), SAv-VIRIP, SAv-VIRIP (reducing, boiled), SAv-JM, SAv-JM (reducing, boiled), SAv-bi-specific, SAv-bi-specific (reducing, boiled).

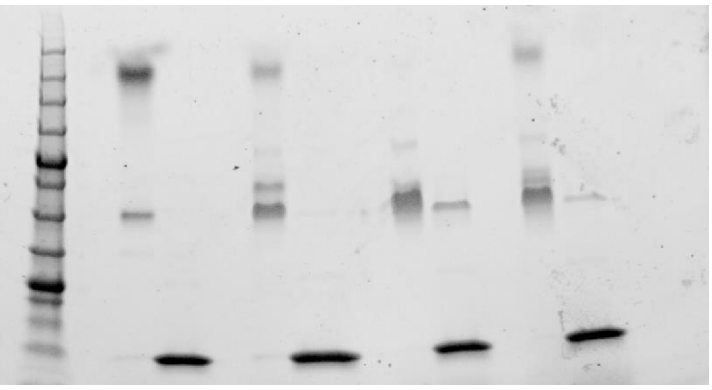

Figure S13: SDS-PAGE analysis of SAv conjugates: Leane from left to right: Leeder, SAv, SAv (reducing conditions), SAv- B-VIR-102C9, SAv-B-VIR-102C9 (reducing conditions), SAv-B-EPI-X4 JM#173-C, SAv-B- EPI-X4 JM#173-C (reducing conditions), SAv-B-VIR-102C9-EPI-X4 JM#173-C, SAv-B-VIR-102C9- EPI-X4 JM#173-C (reducing conditions).

4.2. Assembly of B-VIR-102C9 (5) on Streptavidin

1 mg Streptavidin (10 mg/mL in MilliQ water, 16,7 nmol, 1 equiv.) was added to 900 µL of PB (50 mM, pH 7.4). B-VIR-102C9 (**5**, 0.16 mg, 66,7 nmol, 4 equiv.) was dissolved in 156 µL and added to the protein. The mixture was vortex and shaken at

rt for 15 min. Purification was obtained by using 500 µL Vivaspin ultrafiltration tubes with 10 kDa MWCO and 3x 500 µL PB (50 mM, pH 7.4) spin filtration.

The concentration of the solution was determined as 1.86 mg/mL by Nanodrop absorption measurement at 280 nm and diluted to 25 µM protein concentration for further experiments. Yield was calculated based on protein absorption to be 0.93 mg (93%) of SAv in solution.

4.3. Assembly of B-EPI-X4 JM#173-C (6) on Streptavidin

1 mg Streptavidin (10 mg/mL in MilliQ water, 16,7 nmol, 1 equiv.) was added to 900 µL of PB (50 mM, pH 7.4). B-EPI-X4 JM#173-C (**6**, 0.13 mg, 66.7 nmol, 4 equiv.) was dissolved in 132 µL and added to the protein. The mixture was vortex and shaken at rt for 15 min. Purification was obtained by using 500 µL Vivaspin ultrafiltration tubes with 10 kDa MWCO and 3x 500 µL PB (50 mM, pH 7.4) spin filtration.

The concentration of the solution was determined as 1.95 mg/mL by Nanodrop absorption measurement at 280 nm and diluted to 25 µM protein concentration for further experiments. Yield was calculated based on protein absorption to be 0.90 mg (90%) of SAv in solution.

4.4. Assembly of B-VIR-102C9-EPI-X4 JM#173-C (11) on Streptavidin

1 mg Streptavidin (10 mg/mL in MilliQ water, 16,7 nmol, 1 equiv.) was added to 900 µL of PB (50 mM, pH 7.4). B-VIR-102C9-EPI-X4 JM#173-C (**11**, 0.31 mg, 66.7 nmol, 4 equiv.) was dissolved in 308 µL and added to the protein. The mixture was vortex and shaken at rt for 15 min. Purification was obtained by using 500 µL Vivaspin ultrafiltration tubes with 10 kDa MWCO and 3x 500 µL PB (50 mM, pH 7.4) spin filtration.

The concentration of the solution was determined as 1.57 mg/mL by Nanodrop absorption measurement at 280 nm and diluted to 25 µM protein concentration for further experiments. Yield was calculated based on protein absorption to be 0.82 mg (82%) of SAv in solution.

5. Atomic Force Microscopy (AFM)

Atomic Force Microscopy (AFM) of B-VIR-102C9-EPI-X4 JM#173-C (**11**) 20 µL of a 10 µg/mL protein solution was dropped on a freshly cleaved mica and absorbed for 10 min at room temperature. After subsequent washing with MilliQ H2O and addition of 70 µL MilliQ H2O samples were imaged using liquid tapping mode on a Bruker Dimension FastScan Bio AFM instrument equipped with the ScanAsyst mode with scan rates between 1 and 3 Hz. Images were analyzed by using NanoScope Analysis 1.8 software. The height of the particles were analyzed and the height distribution was determined as shown in the figure and table below by counting 24 particles.

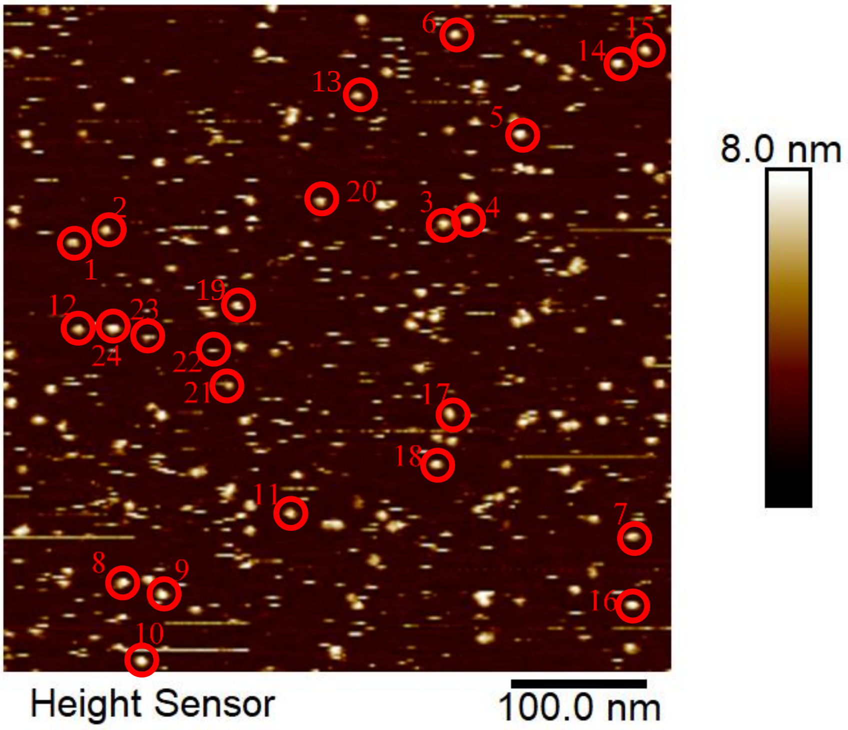

Figure S14: AFM image showing height profile and selection of particles for obtaining height profile

Table S1. Particle height distribution obtained from height profile of AFM image (Figure S14)

**Number**	**Max. Height [nm]**
1	4.496
2	4.533
3	6.145
4	5.623
5	6.366
6	6.195
7	4.647
8	6.115
9	6.074
10	5.980
11	5.591
12	4.789
13	5.483
14	5.934
15	6.016
16	6.405
17	5.886
18	5.867
19	5.231
20	5.012
21	4.515
22	3.057
23	4.927
24	5.982
Average with standard deviation	5.49 ± 0.80

6. Materials and Methods for in vitro Studies

6.1. Cell culture and Primary cells

HEK293T cells were provided and authenticated by the ATCC. TZM-bl cells were provided and authenticated by the NIH AIDS Reagent Program, Division of AIDS, NIAID. HEK293T and TZM-bl cells were maintained in Dulbecco’s modified Eagle medium (DMEM) supplemented with FCS (10%), L-glutamine (2 mM), streptomycin (100 mg/mL) and penicillin (100 U/mL). Cells were cultured at 37°C, 90% humidity and 5% CO2. To obtain PBMCs, Buffy coats were collected from the blood bank (Ulm) and diluted 1:3 with PBS. Ficoll separating solution was overlaid with the diluted blood and centrifuged at 1,600 x g for 20 min without breaks. The white interface layer formed by peripheral blood mononuclear cells (PBMCs) was transferred into a fresh tube and washed twice with PBS. After separation and washing 1 x 10^6 cells/ml were cultured

in supplemented RPMI-1640 and incubated with Human T-Activator CD3/CD28 Dynabeads for 3 days.

6.2 Virus stocks

Virus stocks were generated by transient transfection of HEK293T cells using the calcium-phosphate precipitation method. One day before transfection, 0.8 x 10^6 HEK293T cells were seeded in 6-well plates (Greiner Bio-one, Frickenhausen, Germany). At a confluence of 60-80% cells were used for transfection. For the calcium-phosphate precipitation method, 5 µg DNA was mixed with 13 µl 2 M CaCl2 and the total volume was made up to 100 µl with water. This solution was added drop-wise to 100 µl of 2x HBS. The transfection cocktail was vortexed for 5 sec and added drop-wise to the cells. The transfected cells were incubated for 8-16 h before the medium was replaced by fresh supplemented DMEM. 48 h post transfection, virus stocks were prepared by collecting the supernatant and centrifuging it at 1300 rpm for 3 min.

6.3 TZM-bl infection assay

To determine infectious virus yield, 10,000 TZM-bl reporter cells/well were seeded in 96-well plates and infected with cell culture supernatants (normalized to 100.000 RLU) in triplicates on the following day. Three days p.i., cells were lysed and *β-galactosidase* reporter gene expression was determined using the GalScreen Kit (Applied Bioscience) according to the manufacturer’s instructions with an Orion microplate luminometer (Berthold). Statistical analyses were performed using GraphPad PRISM 9.2 (GraphPad Software). IC50 values were calculated using the Nonlinear regression curve fit tool.

Table S2.

	B-VIR-102C9 (**5**)	SAv-VIR-102C9 (**12**)	B-EPI-X4 JM#173-C (**6**)	SAv-EPI-X4 JM#173-C (**13**)	SAv-VIR-1029C-EPI-X4 JM#173-C (**14**)
IC50 X4 (µM)	1.129	0.025	1.231	0.731	0.026

IC50 R5 (µM)	1.200	0.017	n.d.	n.d.	0.039

6.4 Cell viability

Cell viability was determined by the Cell Titer-Glo 2.0 Cell Viability Assay according to the manufacturer's instructions.

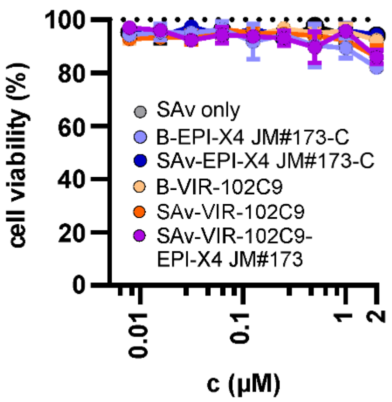

Figure S15: Cell viability of TZM-bl cells treated with increasing amounts of the antiviral peptids. TZM-bl cells were treated with increasing amounts of the indicated compounds and a SAv only control. After 48 h, cell viability was assessed by measuring ATP levels in cells lysates with the commercially available Cell Titer-Glo kit. Concentrations indicate the molarity of the tested biotin conjugated peptides or of the assembled Streptavidin conjugates with four mono- or bispecific peptides respectively. Each dot represents three biological replicates ± SEM.

6.5 Replication kinetics in PBMCs

0.75 million cells were transferred into 96 U well plates, washed twice in PBS and incubated with indicated compounds in RPMI-1640 for 1 hour at 37°C. Then, cells were infected with virus stocks previously generated by transient transfection of HEK293T cells with the respective pro-viral constructs. 16 hours post-infection, cells were washed to remove input virus. At the indicated time points, cells were spun down and ~80% (v/v) of supernatants of the PBMC cultures were aspirated and frozen at -80°C. Medium was replaced with fresh RPMI supplemented with the indicated amounts of the compounds.

6.6 Infectious virus

To determine the infectivity of virions produced in infected human |PBMCs, TZM-bl cells were seeded in 96-well plates at a density of 10,000 cells/well and infected after overnight incubation with the supernatants collected from the PBMC cultures. Three days p.i., viral infectivity was determined using a galactosidase screen kit from Tropix as recommended by the manufacturer. β-Galactosidase activities were quantified as relative light units (RLU) per second with an Orion Microplate luminometer (Berthold).

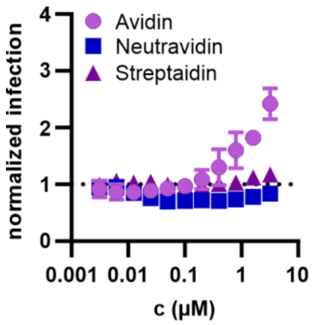

Figure S16: Neutravidin and Streptavidin do not alter HIV-1 infection efficiency. TZM-bl cells were infected with HIV-1 NL4-3 that was preincubated with increasing amounts of the carrier compounds Avidin, Neutravidin or Streptavidin. Three days post infection, a β-galactosidase assay was performed. Each dot represents three biological replicates ± SEM.

.
